# Type 1 Diabetes and the HLA Region: Genetic Association Besides Classical HLA Class II Genes

**DOI:** 10.3389/fgene.2021.683946

**Published:** 2021-06-17

**Authors:** Jana Sticht, Miguel Álvaro-Benito, Stefan Konigorski

**Affiliations:** ^1^Digital Health and Machine Learning Research Group, Hasso Plattner Institute for Digital Engineering, Potsdam, Germany; ^2^Laboratory of Protein Biochemistry, Department of Biology, Chemistry and Pharmacy, Freie Universität Berlin, Berlin, Germany

**Keywords:** GWAS, HLA, type 1 diabetes, UK Biobank, whole exome sequencing

## Abstract

Type 1 diabetes is an autoimmune disease with rising incidence in high-income countries. Genetic and environmental predisposing factors contribute to the etiology of the disease, although their interaction is not sufficiently understood to allow for preventive action. Strongest known associations with genetic variation map to classical HLA class II genes. Because of its genetic complexity, the HLA region has been under-represented in genome-wide association studies, having potentially hindered the identification of relevant associations underlying the etiology of the disease. Here, we performed a comprehensive HLA-wide genetic association analysis of type 1 diabetes including multi-allelic and rare variants. We used high-density whole-exome sequencing data of the HLA region in the large UK Biobank dataset to apply gene-based association tests with a carefully defined type 1 diabetes phenotype (97 cases and 48,700 controls). Exon-based and single-variant association tests were used to complement the analysis. We replicated the known association of type 1 diabetes with the classical *HLA-DQ* gene. Tailoring the analysis toward rare variants, we additionally identified the lysine methyl transferase *EHMT2* as associated. Deeper insight into genetic variation associated with disease as presented and discussed in detail here can help unraveling mechanistic details of the etiology of type 1 diabetes. More specifically, we hypothesize that genetic variation in *EHMT2* could impact autoimmunity in type 1 diabetes development.

## Introduction

Diabetes is a life-threatening condition that requires a tight control of blood sugar levels. The disease is accompanied by health complications and costly therapies throughout life. The estimated prevalence of adults living with diabetes in Europe was 7.3% in 2014 (World Health Organization, 2016). Type 2 diabetes (T2D) is the main form of the disease and preventive action is taken based on well-established risk factors. Type 1 diabetes (T1D) accounts for about 10–15% of the cases (Pociot and Lernmark, 2016), but a 3% annual increase in incidence in high-income countries has been observed over the past decades (World Health Organization, 2016). T1D is usually diagnosed in childhood (Maahs et al., 2010) and preventive strategies have been largely unsuccessful, mainly because of a lack of knowledge about the etiology of this heterogeneous and complex disease.

T1D is an autoimmune disease resulting from the progressive destruction of insulin-producing pancreatic β-cells by the body's own immune system. It is assumed that environmental triggers need to act on a genetically susceptible background to develop disease. On the genetic side, the strongest association with T1D locates to variations in classical HLA class II genes (chromosome 6p21.3), but genetic associations with more than 50 other genes have been identified, most of them related to immune functions (Hu et al., 2015; Pociot and Lernmark, 2016; Pociot, 2017).

HLA proteins present antigenic peptides for T cell surveillance. Genetic variation in the HLA genes influences the peptide pool that can be displayed and recognized to initiate an immune reaction. The high rate of single nucleotide variants (SNVs) in the HLA genes results in altogether 16,755 classical HLA alleles (Kennedy et al., 2017). Based on the strong linkage disequilibrium (LD) in the HLA region, specific combinations of allelic variants form haplotypes. HLA-DR/DQ haplotypes associated with T1D (Erlich et al., 2008) include the allele HLA-DQB1^*^03:02, in which the SNV coding for the D57A variation is responsible for the display of antigenic peptides triggering autoreactive T cell responses, thereby explaining most of the genetic risk for T1D (Hu et al., 2015). However, genetic variation in genes coding for PTPN22 (chromosome 1p13.2) and CTLA4 (chromosome 2q33.2), which have a higher-order role in immune regulation, have also been associated with T1D (Pociot and Lernmark, 2016) indicating that immunity might go astray on a superior level. There is a common agreement that these genes do not account for all the observed heritability of T1D and that the complexity of the HLA region has not been sufficiently accounted for by standard GWAS (Kennedy et al., 2017). Indeed, recent studies on non-classical HLA class II function have shown that these genes could play an important role in T1D (Morgan et al., 2013) and relevant associations of non-HLA genes with T1D might still be hidden in that region.

Here, we used the high-density UK Biobank (UKB) whole exome sequencing (WES) dataset of the HLA region including multi-allelic and rare variants, and employed detailed case-control definitions of T1D. We performed a systematic analysis of the association between genetic variation and T1D on the single-variant, exon- and gene-level in order to identify novel potentially causal protein-coding variants in the HLA region. A deeper understanding of genetic variation associated with T1D can increase our knowledge of the mechanisms underlying autoimmune disease development in general, which in turn can be used to develop preventive, diagnostic or even therapeutic action.

## Methods

### Study Design

UKB is a population-based prospective cohort (Bycroft et al., 2018) assembling genotypic and phenotypic information of 502,536 participants from Great Britain aged 39–70 years at baseline at the time of data retrieval. WES data were available for a subset of 49,997 participants. In order to minimize confounding by relatedness, we restricted the analysis to the 49,025 unrelated participants by excluding up to third-degree relatives. As T1D is a chronic disease with ill-defined onset usually diagnosed in youths, we consider T1D outcome variables as retrospective life-time prevalence.

### Variable Coding and Case-Control Definition

From assessment center visits, self-reported diabetes and T1D diagnosis was available, as well as the age at diagnosis, the information whether participants use insulin medication and whether they started insulin therapy within the first year of diagnosis. Information about main and secondary disease diagnoses were available from hospital admission data in the form of ICD10 codes.

As primary T1D case definition (NDR-defined T1D, 97 cases and 48,700 controls), the epidemiologic definition of the Swedish National Diabetes Register (NDR) (Nationella Diabetesregistret, 2018) was used in all the genetic association analyses: A participant was defined as case, whenever age at diagnosis was <30 years and insulin medication was used. A control was defined as a participant who did not report using insulin medication or reported insulin medication but had an age at diagnosis of ≥30 years. Four further T1D case definitions were constructed for sensitivity analyses: A less stringent definition (“weak,” 302 cases and 35,539 controls) defines a participant as T1D case, if any two combinations of age at diagnosis <30 years, insulin medication used, self-report or ICD10 code for T1D were fulfilled. The more stringent definition (“stringent,” 80 cases and 48,719 controls) includes the use of insulin medication within 1 year of diagnosis besides the NDR criteria. Finally, ICD10-defined (355 cases and 42,232 controls) and self-reported T1D (47 cases and 40,450 controls) case definitions were directly available.

### Preparation of Whole Exome Sequencing Data

The HLA region [bases 29,722,775–33,314,387 (GRCh38/hg38)] on chromosome 6 was extracted from the UKB WES data and included 59,480 SNVs. After filtering out monomorphic variants, singletons and doubletons, and performing standard quality control steps 20,236 SNVs remained. We used an additive coding of the genotypes.

### Statistical Analysis

All the statistical analyses were conducted in R (R Development Core Team, 2010), version 3.6.1. We used the significance level α = 0.05 for hypothesis testing and respective Bonferroni corrections to account for multiple testing in single-variant-, exon-, gene- and allele-level analyses. In all the association tests, we controlled for confounding by relatedness, ethnicity and population structure by restricting the analyses to unrelated participants and by using the top ten genetic principal components (PCs) provided by UKB (Bycroft et al., 2018) as covariates.

In order to test for association between single SNVs and NDR-defined T1D, a logistic regression model was used and evaluated for all 20,236 SNVs separately. In order to model the effects of rare variants jointly to overcome problems of statistical power, region-based tests were performed by using the logistic mixed model sequence kernel association test (SKAT) (Wu et al., 2011). Kernels giving equal weight to all variants (linear) or higher weight to prioritize rare variants (linear-weighted) were computed. SNVs were grouped by gene or exon, resulting in 147 genes and 1,209 exons for analysis. For the allele-based test, we used HLA alleles imputed with the HLA^*^IMP:02 algorithm as provided by UKB (Bycroft et al., 2018), containing 362 classical HLA class I and II alleles. HLA imputation data were available for 48,974 participants from our dataset. A logistic regression analysis using the same statistical model as for the single-SNV analysis was used to test for associations between HLA alleles and NDR-defined T1D.

For more details regarding the study population, coding of diabetes-related variables, UKB data-fields, case-control definitions, preparation of the WES dataset, and statistical analyses, see Supplementary Data.

## Results

### Demographic and Clinical Characteristics of the Dataset

The distribution of demographic and clinical variables in the analyzed dataset of 49,025 unrelated participants of the UKB cohort is shown in Supplementary Table 1. The age distribution at baseline is 39–70 years, the dataset contains slightly more women than men and most of the participants are Caucasians. The descriptive statistics of the diabetes-related variables (Supplementary Table 1) illustrate the challenges of defining T1D cases in large datasets that have not been specifically constructed for the clinical disease phenotype. While 355 individuals ever had a main or secondary ICD10-coded diagnosis for T1D, only 47 self-report to have T1D, with an overlap of only 33 individuals. Thus, in order to reduce misclassification, we employed the epidemiologic definition of T1D used by the Swedish national diabetes register (NDR), which is based on age at diagnosis and insulin medication. This definition has been reported to be in good agreement with clinical diagnoses (Nationella Diabetesregistret, 2018; Rawshani et al., 2018). In our dataset, this results in 97 cases, for which other diabetes-related variables are also in overall agreement with the case definition (Supplementary Table 1). For sensitivity analyses, four alternative T1D definitions were used (see section Methods for details). Differences in demographic and clinical variables among the differentially defined cases are shown in Supplementary Table 2.

### Gene-Based Association Tests

The HLA region contains 148 protein coding genes not only involved in immunity but in various cellular processes (Supplementary Figure 1). In order to test for association of these genes with NDR-defined T1D, we first used the variance-component test SKAT after grouping SNVs in genes. As a WES dataset is used, deep intronic sequences are not included and the association analysis is thus tailored toward coding variants. Without specifically weighting for rare variants (linear kernel) and after Bonferroni correction, 26 genes were significantly associated with NDR-defined T1D (Table 1, *p*-value <3.40·10^−4^). Assuming ~20,000 genes in the human genome (Piovesan et al., 2019), seven genes reach genome-wide significance (*p*-value <2.5·10^−6^). These are six HLA class II genes (*HLA-DQB1, -DQA1, -DRB1* and the paralogues *-DQB2, -DQA2*, and *-DRB5*) in line with known associations of T1D with HLA-DR/DQ haplotypes (Erlich et al., 2008) as well as *PRRT1*. Additionally, the classical *HLA-DRA* and the non-classical *HLA-DO* gene, the peptide transporters *TAP1* and *TAP2* involved in HLA class I antigen processing, the *HLA-DO/TAP2* read-through variant *AL669918.1* and 14 non-HLA-genes are significantly associated after Bonferroni correction. No significant associations were observed with HLA class I genes.

**Table 1:** List of the 26 genes found to be associated with T1D, sorted by *p*-value of the association test using the NDR-defined T1D definition and the linear SKAT kernel after Bonferroni correction for 147 tests.

**Gene**	***p*-value**	***p*-value**	***p*-value**	***p*-value**	***p*-value**	***p*-value**	**n (SNVs)/gene**
	**(linear, NDR)**	**(linear, ICD10)**	**(linear, weak)**	**(linear, stringent)**	**(linear, self-report)**	**(linear-weighted, NDR)**	
PRRT1	**3.96·10**^**−11**^	**1.54·10**^**−06**^	**2.36·10**^**−11**^	**7.39·10**^**−14**^	**2.24·10**^**−17**^	**3.68·10**^**−11**^	33
HLA-DQA1	**6.77·10**^**−11**^	**1.55·10**^**−13**^	**7.94·10**^**−15**^	**4.36·10**^**−10**^	2.02·10^−02^	3.61·10^−04^	159
HLA-DQB1	**6.93·10**^**−11**^	**2.88·10**^**−13**^	**3.69·10**^**−14**^	**3.77·10**^**−11**^	7.54·10^−03^	6.77·10^−03^	331
HLA-DRB5	**7.72·10**^**−08**^	**5.51·10**^**−11**^	**1.58·10**^**−11**^	**7.44·10**^**−10**^	**3.36·10**^**−04**^	**1.72·10**^**−04**^	436
HLA-DQA2	**8.37·10**^**−08**^	8.70·10^−04^	**3.63·10**^**−05**^	**1.71·10**^**−07**^	1.09·10^−02^	2.22·10^−01^	120
HLA-DRB1	**3.24·10**^**−07**^	**1.10·10**^**−07**^	**7.21·10**^**−08**^	**6.16·10**^**−07**^	6.15·10^−02^	**4.08·10**^**−05**^	579
HLA-DQB2	**1.79·10**^**−06**^	1.25·10^−03^	**3.46·10**^**−05**^	**2.76·10**^**−07**^	1.20·10^−02^	**1.94·10**^**−06**^	186
HSPA1A	**4.93·10**^**−06**^	**1.59·10**^**−07**^	**5.98·10**^**−09**^	**1.02·10**^**−06**^	4.37·10^−03^	1.20·10^−01^	71
TSBP1	**1.17·10**^**−05**^	**8.01·10**^**−07**^	**1.28·10**^**−08**^	**7.42·10**^**−07**^	3.99·10^−03^	5.11·10^−02^	216
HLA-DRA	**1.18·10**^**−05**^	**1.79·10**^**−05**^	**1.30·10**^**−06**^	**4.99·10**^**−06**^	5.82·10^−02^	6.39·10^−02^	52
ABHD16A	**1.31·10**^**−05**^	**7.71·10**^**−07**^	**2.51·10**^**−07**^	**9.83·10**^**−07**^	3.11·10^−02^	1.32·10^−01^	197
CLIC1	**1.81·10**^**−05**^	**5.00·10**^**−06**^	**8.11·10**^**−07**^	**5.09·10**^**−06**^	1.47·10^−02^	5.37·10^−03^	69
AL669918.1	**2.46·10**^**−05**^	5.29·10^−03^	**3.58·10**^**−05**^	**1.52·10**^**−04**^	4.74·10^−02^	2.60·10^−03^	387
VWA7	**2.61·10**^**−05**^	**1.15·10**^**−06**^	**1.19·10**^**−07**^	**8.07·10**^**−06**^	9.27·10^−02^	1.43·10^−03^	233
MSH5	**2.76·10**^**−05**^	**2.11·10**^**−06**^	**1.60·10**^**−07**^	**5.01·10**^**−06**^	4.77·10^−02^	2.95·10^−03^	236
HLA-DOB	**2.82·10**^**−05**^	1.32·10^−03^	**2.69·10**^**−05**^	**2.09·10**^**−04**^	6.23·10^−02^	**1.32·10**^**−04**^	83
TAP2	**3.54·10**^**−05**^	7.76·10^−03^	**5.04·10**^**−05**^	**1.90·10**^**−04**^	4.52·10^−02^	5.85·10^−03^	328
MSH5-SAPCD1	**3.79·10**^**−05**^	**2.54·10**^**−06**^	**1.83·10**^**−07**^	**6.19·10**^**−06**^	4.18·10^−02^	5.04·10^−03^	283
PRRC2A	**5.22·10**^**−05**^	**1.81·10**^**−08**^	**8.98·10**^**−10**^	**3.69·10**^**−06**^	4.61·10^−02^	5.14·10^−01^	517
C6orf47	**6.35·10**^**−05**^	**6.39·10**^**−06**^	**1.68·10**^**−07**^	**1.80·10**^**−05**^	2.12·10^−03^	1.00·10^+00^	42
PSMB9	**7.98·10**^**−05**^	6.10·10^−03^	7.19·10^−04^	**1.79·10**^**−04**^	3.01·10^−01^	1.31·10^−01^	315
BRD2	**1.72·10**^**−04**^	1.54·10^−02^	2.44·10^−03^	**2.90·10**^**−05**^	4.79·10^−04^	1.34·10^−01^	288
TAP1	**1.82·10**^**−04**^	5.72·10^−03^	1.01·10^−03^	**3.22·10**^**−04**^	1.88·10^−01^	2.72·10^−01^	201
CYP21A2	**1.86·10**^**−04**^	**3.85·10**^**−06**^	**7.91·10**^**−09**^	**7.48·10**^**−05**^	2.24·10^−02^	2.43·10^−02^	230
LSM2	**2.01·10**^**−04**^	**5.34·10**^**−05**^	**6.26·10**^**−06**^	**3.90·10**^**−05**^	6.21·10^−02^	3.83·10^−01^	39
BTNL2	**2.28·10**^**−04**^	6.36·10^−03^	6.12·10^−04^	**9.81·10**^**−05**^	2.76·10^−01^	2.02·10^−01^	172
AL662899.3	3.77·10^−04^	**2.05·10**^**−05**^	**6.32·10**^**−06**^	**3.24·10**^**−05**^	4.52·10^−02^	2.23·10^−01^	277
BAG6	4.95·10^−04^	**1.10·10**^**−06**^	**5.94·10**^**−07**^	**1.25·10**^**−04**^	1.16·10^−01^	1.25·10^−01^	276
DDAH2	5.74·10^−04^	3.98·10^−02^	1.06·10^−02^	**1.79·10**^**−04**^	**6.30·10**^**−05**^	3.50·10^−04^	43
ATP6V1G2	7.55·10^−04^	**1.09·10**^**−04**^	**2.23·10**^**−05**^	1.23·10^−03^	6.00·10^−02^	9.75·10^−01^	47
DXO	9.45·10^−04^	1.19·10^−03^	**2.85·10**^**−04**^	**1.52·10**^**−05**^	4.09·10^−02^	7.69·10^−03^	89
CFB	9.56·10^−04^	**5.49·10**^**−05**^	**1.50·10**^**−05**^	5.17·10^−04^	5.91·10^−02^	1.01·10^−03^	169
TNXB	1.33·10^−03^	**3.22·10**^**−04**^	**1.49·10**^**−05**^	**2.77·10**^**−04**^	1.59·10^−02^	**5.37·10**^**−05**^	1,022
ATP6V1G2-DDX39B	1.62·10^−03^	4.28·10^−04^	**5.12·10**^**−05**^	2.20·10^−03^	4.74·10^−02^	2.04·10^−01^	119
AGER	1.82·10^−03^	**1.73·10**^**−06**^	**1.17·10**^**−06**^	4.58·10^−03^	1.95·10^−01^	1.53·10^−01^	113
MICB	2.50·10^−03^	7.87·10^−04^	**2.52·10**^**−04**^	2.02·10^−03^	6.11·10^−03^	2.66·10^−01^	137
DDX39B	2.72·10^−03^	8.82·10^−04^	**9.44·10**^**−05**^	3.47·10^−03^	5.28·10^−02^	1.33·10^−01^	94
NOTCH4	3.78·10^−03^	3.66·10^−04^	**7.71·10**^**−05**^	6.85·10^−03^	3.95·10^−01^	1.18·10^−03^	404
VARS1	5.56·10^−03^	**3.40·10**^**−05**^	**2.00·10**^**−05**^	8.50·10^−04^	1.01·10^−01^	9.05·10^−01^	277
ATF6B	5.97·10^−03^	6.73·10^−04^	**2.62·10**^**−04**^	3.32·10^−03^	9.56·10^−02^	5.97·10^−01^	201
GPANK1	6.47·10^−03^	**1.14·10**^**−04**^	**5.05·10**^**−05**^	7.12·10^−04^	8.25·10^−02^	9.01·10^−02^	106
PPT2	7.63·10^−03^	**1.38·10**^**−05**^	**1.86·10**^**−05**^	1.69·10^−02^	2.01·10^−01^	6.03·10^−01^	88
PPT2-EGFL8	1.84·10^−02^	**2.23·10**^**−04**^	3.46·10^−04^	3.65·10^−02^	1.61·10^−01^	8.43·10^−02^	203
GPSM3	1.86·10^−02^	**3.13·10**^**−05**^	**1.13·10**^**−04**^	3.97·10^−02^	5.81·10^−01^	1.17·10^−01^	57
RNF5	1.91·10^−02^	**1.62·10**^**−05**^	**4.38·10**^**−05**^	3.36·10^−02^	4.73·10^−01^	1.32·10^−01^	36
AL645922.1	2.39·10^−02^	4.77·10^−04^	1.06·10^−03^	1.48·10^−02^	8.50·10^−02^	**2.07·10**^**−04**^	376
EHMT2	2.84·10^−02^	3.57·10^−03^	2.53·10^−03^	2.32·10^−02^	1.95·10^−01^	**1.27·10**^**−07**^	296
ZNRD1	3.09·10^−02^	**1.60·10**^**−04**^	2.51·10^−03^	5.60·10^−02^	2.38·10^−01^	1.28·10^−01^	44
NELFE	5.03·10^−01^	1.47·10^−01^	2.66·10^−01^	3.79·10^−01^	9.13·10^−01^	**9.07·10**^**−06**^	91

*Additionally, results using the linear SKAT kernel with the four alternative T1D definitions (ICD10, weak, stringent, self-reported) as well as using the linear-weighted SKAT kernel with the NDR-defined T1D definition are also shown. The number of SNVs per analyzed gene is given in the last column. Significant p-values after Bonferroni correction (<0.05/147 i.e., 3.40·10^−4^) are shown in bold. The gene ID as well as start and end position is given in Supplementary Table 3*.

**Figure 1:**
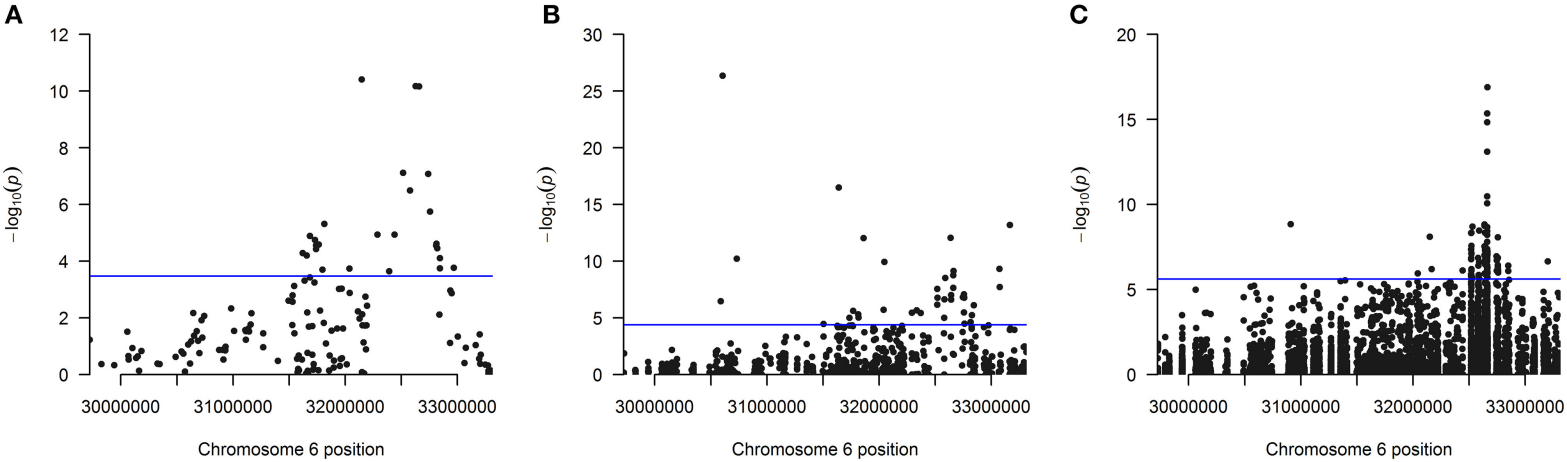
**(A)** Manhattan plot of all 147 analyzed genes showing the position of the 26 genes found significantly associated after Bonferroni correction using the linear kernel SKAT test (see also Table 1). **(B)** Manhattan plot for all 1,209 analyzed exons showing the position of the 40 exons found significantly associated after Bonferroni correction using the linear kernel SKAT test (see also Table 2). **(C)** Manhattan plot for all 20,236 analyzed SNVs showing the position of the 92 SNVs found significantly associated after Bonferroni correction using logistic regression (see also Supplementary Table 6). The line indicates the significance cutoff after Bonferroni correction that is **(A)** [–log (0.05/147) = 3.47], **(B)** [–log (0.05/1209) = 4.38] and **(C)** [–log (0.05/20236) = 5.61]. A circle is drawn for every SNV or at every gene's or exon's start position.

The distribution of the 26 significantly associated genes in the HLA region on chromosome 6 is shown in the Manhattan plot in Figure 1A, illustrating that the associated genes cluster in a defined region. The quantile-quantile (QQ)-plot in Supplementary Figure 2A clearly shows many small *p*-values (median *p*-value 0.05), likely resulting from the extensive LD in the HLA region (de Bakker et al., 2006). Repeating the analysis with a dataset containing only common variants (MAF > 0.01) led to the exclusion of the genes *PRRT1, IER3, LTB, LY6G6C, DDAH2, NEU1* from the analysis, as they now have <2 SNVs per gene. There was no further effect on the result, indicating that the common variants dominated the outcome (Supplementary Table 4 and Supplementary Figure 2B).

**Figure 2:**
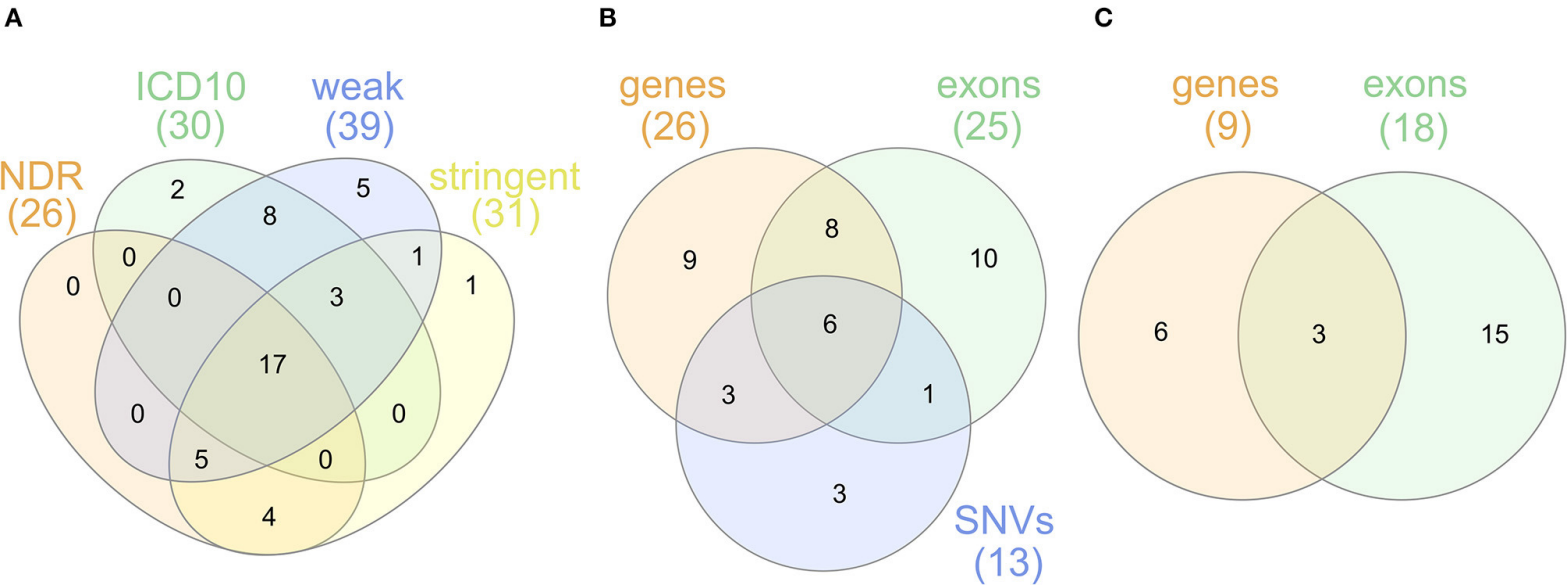
**(A)** Venn diagram for genes found to be significantly associated with the T1D definitions NDR, ICD10, weak and stringent using the linear SKAT model. **(B)** Venn diagram for genes found to be significantly associated with NDR-defined T1D in the single-SNV analysis and the gene- or exon-based test using the linear SKAT model. Six genes (*HLA-DQA1, -DQB1, -DRB1, -DQA2, -DQB2, -DRB5*) were identified in all tests, eight genes (*AL669918.1, CLIC1, CYP21A2, HLA-DOB, HSPA1A, TAP2, TSBP1*, and *VWA7*) in the gene- and exon-based SKAT tests, and three genes (*HLA-DRA, PRRT1*, and *TAP1*) in the gene-based SKAT and the single-SNV analysis. *TNXB* was identified in the exon-based and the single-SNV analysis. **(C)** Venn diagram for genes found to be significantly associated with NDR-defined T1D in the gene- or exon-based test using the linear-weighted SKAT model. Three genes (*HLA-DOB, TNXB*, and *EHMT2*) were identified in both tests.

For sensitivity analyses, the gene-based SKAT test using the linear model was repeated with the four alternative T1D definitions (Table 1). By using the self-reported T1D definition, only three significantly associated genes were observed likely resulting from an impact of low case counts (Supplementary Table 2) on statistical power. In contrast, the four other T1D case definitions detected an overlap of 17 genes significant after Bonferroni correction (Figure 2A) with *PRRT1, HLA-DQB1, -DQA1, -DRB1*, and -*DRB5* showing genome-wide significance.

### Exon-Based Association Tests

Next, 1,209 exons were analyzed in exon-based SKAT thereby focusing on the impact of protein-coding variants. In total, 40 exons belonging to 25 genes were significantly associated with NDR-defined T1D using the linear kernel SKAT test after Bonferroni correction (Table 2, *p*-value <4.14·10^−5^). Assuming ~550,000 exons in the human genome (Piovesan et al., 2019), 15 exons reach genome-wide significance (*p*-value <9·10^−8^). The distribution of associated exons in the analyzed HLA region is shown in the Manhattan plot in Figure 1B. Again, small *p*-values dominate the shape of the QQ-plot (Supplementary Figure 2D), although large *p*-values are overrepresented (median *p*-value 0.71).

**Table 2:** List of exons found to be significantly associated with NDR-defined T1D using the linear as well as the linear-weighted SKAT kernel, after Bonferroni correction for 1,209 tests.

**Exon ID**	**Gene**	**Exon**	***p*-value (linear)**	***p*-value (linear-weighted)**	**n (SNVs)/exon**
ENSE00001707124	PPP1R10	exon8	**4.46·10**^**−27**^	**4.46·10**^**−27**^	2
ENSE00003635259	BAG6	exon16	**3.19·10**^**−17**^	**3.18·10**^**−17**^	3
ENSE00001656329	COL11A2	exon55	**6.55·10**^**−14**^	**5.73·10**^**−14**^	3
ENSE00001766857	HLA-DQA1	exon1	**8.77·10**^**−13**^	3.64·10^−02^	16
ENSE00001836503	SLC44A4	exon21	**9.27·10**^**−13**^	**9.10·10**^**−13**^	6
ENSE00003786549	FLOT1	exon9	**6.23·10**^**−11**^	**6.25·10**^**−11**^	4
ENSE00001792039	TNXB	exon30	**1.20·10**^**−10**^	**1.20·10**^**−10**^	11
ENSE00001619685	HLA-DPA1	exon1	**4.98·10**^**−10**^	**4.96·10**^**−10**^	14
ENSE00003421994	HLA-DQB1	exon2	**7.74·10**^**−10**^	2.33·10^−02^	71
ENSE00001596914	HLA-DQB1	exon3	**1.84·10**^**−09**^	3.73·10^−01^	25
ENSE00001930619	HLA-DRB1	exon1	**3.11·10**^**−09**^	9.00·10^−04^	69
ENSE00001911406	HLA-DPB1	exon1	**2.01·10**^**−08**^	**1.92·10**^**−08**^	8
ENSE00003562109	HLA-DQB1	exon4	**2.34·10**^**−08**^	4.93·10^−01^	13
ENSE00001641881	HLA-DRB5	exon3	**2.86·10**^**−08**^	5.82·10^−05^	50
ENSE00001703275	HLA-DQB2	exon4	**8.62·10**^**−08**^	1.76·10^−02^	15
ENSE00003658074	HLA-DQA1	exon3	**9.78·10**^**−08**^	5.99·10^−01^	24
ENSE00001614414	HLA-DQA2	exon4	**1.56·10**^**−07**^	9.10·10^−02^	22
ENSE00001723559	HLA-DQB2	exon3	**1.76·10**^**−07**^	6.27·10^−02^	26
ENSE00001768120	HLA-DRB5	exon2	**1.77·10**^**−07**^	1.20·10^−01^	107
ENSE00001731226	HLA-DRB1	exon2	**2.43·10**^**−07**^	1.23·10^−01^	104
ENSE00001465231	HLA-DQA1	exon4	**2.51·10**^**−07**^	1.14·10^−02^	35
ENSE00003589061	ABCF1	exon15	**3.55·10**^**−07**^	**3.56·10**^**−07**^	2
ENSE00001715371	HLA-DRB5	exon4	**6.99·10**^**−07**^	2.41·10^−01^	13
ENSE00001646872	PSMB8	exon1	**7.82·10**^**−07**^	**7.73·10**^**−07**^	8
ENSE00003839368	TNXB	exon44	**1.95·10**^**−06**^	4.41·10^−02^	16
ENSE00003843744	CYP21A2	exon10	**2.05·10**^**−06**^	1.19·10^−01^	42
ENSE00003744412	TSBP1	exon16	**2.27·10**^**−06**^	2.09·10^−01^	2
ENSE00003555889	VWA7	exon8	**2.50·10**^**−06**^	**2.66·10**^**−06**^	8
ENSE00001663669	HLA-DQB2	exon5	**3.35·10**^**−06**^	5.00·10^−05^	45
ENSE00003725416	TSBP1	exon32	**3.58·10**^**−06**^	3.27·10^−01^	13
ENSE00003739283	TSBP1	exon1	**3.78·10**^**−06**^	1.09·10^−02^	9
ENSE00001690505	HSPA1A	exon1	**4.93·10**^**−06**^	1.20·10^−01^	71
ENSE00003465858	AL669918.1	exon11	**5.86·10**^**−06**^	**3.90·10**^**−05**^	10
ENSE00003685114	TAP2	exon11	**5.86·10**^**−06**^	**3.90·10**^**−05**^	10
ENSE00003703225	HSPA1A	exon2	**9.46·10**^**−06**^	2.27·10^−01^	32
ENSE00001801024	HLA-DRB1	exon3	**1.03·10**^**−05**^	8.41·10^−04^	61
ENSE00001858405	CLIC1	exon1	**1.04·10**^**−05**^	2.63·10^−04^	13
ENSE00001625708	HLA-DOB	exon1	**2.41·10**^**−05**^	**1.58·10**^**−05**^	8
ENSE00001775810	HLA-DQB2	exon2	**3.41·10**^**−05**^	1.88·10^−01^	9
ENSE00001691563	MICB	exon3	**3.60·10**^**−05**^	2.94·10^−01^	14
ENSE00001727868	PSMB9	exon5	4.15·10^−03^	**2.18·10**^**−05**^	10
ENSE00003580154	EHMT2	exon2	1.61·10^−01^	**7.87·10**^**−06**^	9
ENSE00003463002	VARS2	exon26	7.16·10^−01^	**1.68·10**^**−08**^	8
ENSE00003569101	SKIV2L	exon8	7.73·10^−01^	**4.39·10**^**−11**^	8

*Results are sorted by p-value of the association test using the linear SKAT kernel. Significant p-values after Bonferroni correction (<0.05/1,209 i.e., <4.14·10^−5^) are shown in bold. The number of analyzed SNVs per exon is given in the last column. Start and end position of the exons are given in Supplementary Table 5*.

Found with genome-wide significance in both the gene- and exon-based tests were only the genes *HLA-DQB1, -DQA1, -DRB1* and the paralogues *-DQB2* and *-DRB5*, in line with previous knowledge. Significant after Bonferroni correction in both the exon- and gene-based test were additionally the classical *HLA-DQA2*, the non-classical *HLA*-*DOB* gene, *TAP2, AL669918*.1, *CLIC1, CYP21A2, HSPA1A, TSBP1*, and *VWA7*.

### Role of Rare Variants in Region-Based Tests

In order to unravel the association of NDR-defined T1D with rare genetic variants, we repeated the gene-based SKAT increasing the weight of rare (MAF <0.01) and low-frequency variants (MAF 0.01–0.05) in the association test (linear-weighted kernel) (Wu et al., 2011). Most genes (21 out of 26) detected to be significant with the linear kernel showed no significant association when using the linear-weighted kernel (Table 1), indicating that their association is dominated by common variants. Four genes (*TNXB, AL645922*.1, *EHMT2*, and *NELFE*) were only significantly associated with NDR-defined T1D using the linear-weighted model, implying a role of rare or low-frequency variants. In the attempt to determine protein-coding regions that might account for these associations, the exon-based test was repeated using linear-weighted SKAT. This removed the signal of 26 out of the 40 associations found with the linear kernel (Table 2). Four exons belonging to the genes *PSMB9, EHMT2, VARS2*, and *SKIV2L* were significantly associated with NDR-defined T1D using the linear-weighted but not the linear SKAT model (Table 2), indicating that rare variants might account for their association.

### Single-SNV Association Analysis

Region-based association tests aggregate single SNVs. In case that common variants dominate the association, a single-SNV logistic regression analysis can identify SNVs responsible for the association of a genetic region. Here, the single-SNV logistic regression analysis of the 20,236 SNVs in the WES data of the HLA region and NDR-defined T1D resulted in 92 significantly associated SNVs in 13 genes after Bonferroni correction (Supplementary Table 6, *p*-value <2.47·10^−6^). Twenty-eight SNVs belonging to the genes *HLA-DQB1, -DQA1, -DRB1, -DQB2, -DRB5, GTF2H4, PRRT1* are genome-wide significant with a *p*-value <5·10^−8^ and all of them reach the suggestive level of statistical significance (*p*-value <1·10^−5^). The distribution of these associated SNVs in the analyzed HLA region is shown in the Manhattan plot in Figure 1C, indicating the strong involvement of the HLA-DR/DQ region: 85 significantly associated SNVs locate to *HLA*-*DQB1*, -*DQA1*, -*DRB1*, -*DRA*, and their paralogues -*DQA2*, -*DQB2*, and -*DRB5*. The most significant SNV codes for the HLA-DQB1 D57A variation, the leading risk variant for T1D (Hu et al., 2015). In contrast to that, no significant association was detected with single SNVs coding for variation in HLA-DRB1 amino acid positions β13 and β71 that had also been described to confer strong risk for T1D (Hu et al., 2015). Possibly, relevant SNVs remain below the significance cut-off in our analysis, as for example two SNVs (6:32584354:C:A and 6:32584355:T:A) coding for variation in HLA-DRB1 β13 show protective odds ratios [OR (95%CI) = 0.53 (0.29; 0.96) and 0.53 (0.29; 0.95)] but non-significant *p*-values (3.79·10^−2^ and 3.42·10^−2^).

Odds ratios for all the significantly associated SNVs within *HLA-DQB1*, -*DQA1*, -*DRB1*, -*DRA*, and -*DRB5* genes show a clearly increased chance to have T1D as compared to the reference nucleotide (Supplementary Table 6). Some of the SNVs in the paralogues *HLA*-*DQA2* and -*DQB2* show protective odds ratios.

Only seven of the significantly associated SNVs locate to non-HLA proteins and code for variation in intronic regions of *TAP1, GTF2H4, PRRT1, SLC39A7*/*RXRB* and in protein coding regions of *EGFL8* (R69C) and *TNXB* (E4051K). However, the low MAF and wide confidence intervals of the odds ratios of the significant SNVs in *GTF2H4, SLC39A7*/*RXRB, EGFL8*, and *TNXB* imply that these associations have to be interpreted with care (Supplementary Table 6). The shape of the QQ-plot (Supplementary Figure 2E) is dominated by high *p*-values (median 0.98), resulting from the fact that most SNVs in the dataset are rare variants (MAF <0.01) (see also Supplementary Figure 2F).

### Combined Results

The overlap of genes associated in the single-variant as well as the gene- and exon-based tests using the linear kernel is illustrated in the Venn diagram in Figure 2B. The six genes associated after Bonferroni correction in all three tests are the classical HLA class II genes *HLA-DQA1*, -*DQB1, -DRB1* and their paralogues *-DQA2, -DQB2, -DRB5*. The classical *HLA-DRA* gene, the non-classical *HLA-DO* and 10 non-HLA genes are associated in two of the association tests. Focusing on rare variants, the genes *HLA-DO, TNXB*, and *EHMT2* were found associated both in the gene- and exon-based tests (Figure 2C), but only *EHMT2* was exclusively associated using the linear-weighted SKAT kernel in both tests, indicating that rare protein-coding variation might lead to the association.

## Discussion

In this study, we used UKB WES data to investigate genetic associations in the HLA region with T1D in detail. By using gene- and exon-based as well as single-variant tests, we could confirm known associations of T1D with classical HLA class II genes, and we identified new candidate genes for independent associations.

In our UKB dataset, 5.9% of the participants self-reported to have any type of diabetes (Supplementary Table 1). This is consistent with a diabetes prevalence of 7.3% in Europe (World Health Organization, 2016) in combination with the healthy cohort effect seen in UKB (Fry et al., 2017). However, instead of expected 10–15% (Rewers and Ludvigsson, 2016), only 3.3% of the diabetes cases have NDR-defined T1D. Some true T1D cases diagnosed later in life (Thomas et al., 2018) might be excluded in the NDR definition due to the age criterion. However, the average age at diagnosis in the alternative case definitions is unexpectedly high (Supplementary Table 2) suggesting that the NDR definition better differentiates T1D from T2D. Underlying our choice of the NDR case definition is also that, given the massive case-control imbalance in our dataset, misclassifying a control as a case would be more harmful than misclassifying a case as a control. This goes hand in hand with lower case counts, resulting in less statistical power than in more targeted epidemiologic studies, despite the large size of the UKB cohort. To make sure that the identified associations are not due to an arbitrary case-control definition, we investigated multiple T1D definitions, corroborating the results.

The known association of T1D with the HLA-DR/DQ haplotype was robustly replicated here, as seven classical HLA class II genes were detected in at least two of the applied association tests (Figure 2B), most of them with genome-wide significance in all three tests (Tables 1, 2, Supplementary Table 6) and also with alternative T1D definitions (Table 1). In line with previous results (Hu et al., 2015; Bycroft et al., 2018), an allele-based association test applied on our dataset identified the HLA allele DQB1^*^0302 as most strongly associated with NDR-defined T1D (Supplementary Table 7). A SNV (6:32664911:T:G) coding for the D57A substitution in HLA-DQB1 has been found enriched in T1D patients already before the era of GWAS (Todd et al., 1987). Its genetic association with T1D has later been inferred from imputed HLA alleles (Hu et al., 2015), but a direct association has neither been reported in the GWAS Catalog (Buniello et al., 2019), nor in UKB repositories (Zhou et al., 2018; McInnes et al., 2019; Zhao et al., 2020). Here, we directly detect this multi-allelic SNV to be associated with NDR-defined T1D. This illustrates that relevant common SNVs in the HLA region are often excluded from GWAS most likely by standard quality control procedures as filtering for Hardy-Weinberg-equilibrium (HWE), despite that deviations from HWE are evolutionary common in the HLA region (Kennedy et al., 2017).

Another feature complicating the analysis of the data on protein level is the high rate of polymorphism in the HLA region, as the functional impact of a single SNV can depend on additional variation in the same codon. For example, our dataset contains six SNVs within the codon for HLA-DRB1 β13 that could theoretically result in 24 different combinations coding for 10 different amino acids at that position. This could well explain the lack of significant associations for the individual SNVs here, although variation at amino acid position HLA-DRB1 β13 has been shown to be associated with T1D (Hu et al., 2015).

Besides the clear role of classical HLA alleles for T1D, recent work has shown the impact of SNVs in non-classical HLA genes on their function in antigen presentation (Sirota et al., 2009; Alvaro-Benito et al., 2018; Graves et al., 2020) and animal models suggested a role in susceptibility to T1D (Yi et al., 2010; Morgan et al., 2013). Although we analyzed WES data, we did not detect SNVs or exons coding for the mature protein domains of the non-classical HLA genes *HLA-DM* and -*DO* as associated with NDR-defined T1D. The low minor allele frequencies (MAFs) of SNVs in these genes (Alvaro-Benito et al., 2016) in combination with the low case counts likely resulted in a lack of statistical power. This might also apply to the V142I variation in HLA-DM that is characteristic for the HLA-DMA^*^01:02 allele shown to have a protective association with T1D (Cucchi-Mouillot et al., 1998). In our dataset, this variant was not significantly associated after Bonferroni correction but showed protective odds ratios [OR (95% CI) = 0.52 (0.30; 0.92), *p*-value = 2.3·10^−2^]. We did however detect the gene *HLA-DO* and its exon1 to be associated with T1D. As *HLA-DO* exon1 codes for the signal peptide, the variation might result in impaired protein sorting leading to a dosage effect on protein level.

Besides the HLA genes, we also identified the non-HLA genes *AL669918*.1, *CLIC1, CYP21A2, HSPA1A, PRRT1, TAP1, TAP2, TNXB, TSBP1*, and *VWA7* to be associated with NDR-defined T1D in two of our association tests (Figure 2B) and with alternative phenotype definitions (Table 1). *EHMT2* was found associated in addition, when focusing on rare variants. Associations of *AL669918.1, TAP2* and *TSBP1*-*AS1* with T1D have been reported previously (Tomer et al., 2015; Buniello et al., 2019). SNVs in the genes *CLIC1, CYP21A2, HSPA1A, PRRT1, TNXB, VWA7*, and *EHMT2* have only been reported in association with T1D in UKB repositories (Zhou et al., 2018; McInnes et al., 2019; Zhao et al., 2020). As mentioned above, the strong LD in the HLA region might lead to associations dependent on HLA-DR/DQ loci. Supplementary Figure 3 illustrates that long-range LD in the ~1.2 Mb region harboring these genes is detectable, but strongest LD occurs in much smaller blocks. Indeed, conditional on a *HLA-DQB1* SNV (6:32660935:C:T), *HLA-DO, TAP1, TAP2, AL669918.1* in the vicinity of HLA-DR/DQ as well as *PRRT1* were not associated anymore in the gene-based test. The other genes' association appears to be independent from the HLA-DR/DQ locus.

In terms of biological plausibility, some of the associated genes have already been linked to diabetes. CLIC1, a nuclear chloride ion channel, has been suggested to be involved in pancreatic β-cell mass expansion during pregnancy (Horn et al., 2016) and to be a target of the T2D drug metformin (Gritti et al., 2014). The chaperone HSPA1A (Hsp70) has been implicated in selecting pro-insulin antigens for HLA presentation (Kolb and Burkart, 2019). A top candidate for a functionally relevant association with T1D is EHMT2 that has been shown to regulate T cell development and differentiation (Scheer and Zaph, 2017) in its function as methyltransferase that di-methylates lysine 9 of histone 3 (H3K9), a signal for transcriptional repression. Here, we found *EHMT2* exon2 significantly associated with T1D when focusing on rare variants. Exon2 harbors the SNV (6:31896761:G:A; MAF = 0.02; coding for S58F) with the lowest *p*-value [2.53·10^−05^, OR = 3.40 (1.92;6.00), heterozygous in 13 out of 97 T1D cases] within the *EHMT2* gene. This low-frequency SNV has been found to be associated with T2D independent of *HLA-DQA1* (Bonas-Guarch et al., 2018). Here, we confirm that variation in *EHMT2* is not in LD with HLA-DR/DQ loci (Supplementary Figures 3, 4). The association with T2D might be explained by EHMT2's function in insulin-dependent regulation of transcription (Arai et al., 2015). In case of T1D, another pathway might be involved: Inhibition of EHMT2 has been shown to enhance CTLA4 and FOXP3 expression in regulatory T cells (Ding et al., 2019), both are markers of regulatory T cell function needed to maintain tolerance and prevent autoimmunity. As CTLA4 is known to be associated with T1D (Pociot and Lernmark, 2016), alterations in EHMT2 expression and/or function owing to natural variations may as well be linked to autoimmunity.

In summary, using a combination of single-SNV, exon- and gene-based analyses using WES data of the HLA region, we replicate known associations of HLA class II genes with T1D and suggest *EHMT2* as candidate for a functionally relevant association. We believe that this study justifies a follow-up in functional analyses of EHMT2 protein variants. Furthermore, an analysis of whole genome sequencing data of the HLA region, as soon as available in UK Biobank, could gain additional insight into a potential association with non-coding SNVs. Genetic variations represent unpreventable causal components of disease development but they can improve our understanding of the underlying mechanism. This knowledge can translate into the identification of drug targets (Okada et al., 2014). In case of T1D, it might help to understand if and how the destruction of insulin-producing β-cells could possibly be stopped or prevented.

## Data Availability Statement

Publicly available datasets were analyzed in this study. This data belongs to the UK Biobank and can be accessed upon application at: https://ukbiobank.ac.uk/register-apply/.

## Author Contributions

JS, MÁ-B, and SK designed the research, interpreted the data, and wrote the manuscript. JS and SK performed the biostatistical analyses. All authors contributed to the article and approved the submitted version.

## Conflict of Interest

The authors declare that the research was conducted in the absence of any commercial or financial relationships that could be construed as a potential conflict of interest.
