# Supplementary material for: Type 1 Diabetes and the HLA Region: Genetic Association Besides Classical HLA Class II Genes
Source: Front Genet. 2021 Jun 17;12:683946. doi: 10.3389/fgene.2021.683946 (PMC8248358; doi:10.3389/fgene.2021.683946)
Supplement: Supplementary file 1 [file Data_Sheet_1.PDF]

Supplementary Material

1 Supplementary Data

1.1 Study design

UK Biobank (UKB) participants volunteered to participate in a center-based recruitment strategy. A relatively high relatedness (Bycroft et al., 2018), a low participation rate of contacted individuals (5.5%) as well as a "healthy participants" effect have been observed (Fry et al., 2017), so that UKB participants are not representative of the UK population (Keyes and Westreich, 2019).

Data was extracted from the UKB resource in August 2019, at that time including 502,536 study participants, with whole exome sequencing (WES) data for a subset of 49,997 participants. This subset of participants is representative of the whole cohort with respect to age, sex, and ethnicity, but predominantly includes participants with complete phenotype information and is enriched for participants with asthma (Van Hout et al., 2019). Pairs of participants with up to 3rd-degree relatedness as based on kinship coefficients computed from the genetic data were excluded from the analysis, resulting in 49,025 unrelated participants.

1.2 Variable coding and case-control definition

The UKB Data-Field IDs are given in brackets behind the variables used.

The variables age (ID 21022), sex (as determined based on genotyping, ID 22001) and ethnicity (individuals were defined as Caucasians that self-reported to be "white" and were confirmed to be of Caucasian ancestry by principle component analysis of their genetic data, ID 22006) were used as provided by UKB.

For diabetes-related variables, the answers "don't know" and "prefer not to answer" were coded as missing values and in case follow-up recordings existed, they were combined. The information for any type of diabetes ("yes" or "no", ID 2443) was combined from up to three different time points in a way that "yes" was coded for every participant who ever answered "yes", and "no" was coded for anyone, who never answered "yes" and at least once answered "no". The information if insulin therapy was started within the first year of diagnosis (ID 2986) was coded in the same way. Two questions asked whether insulin was used as medication (ID 6177, ID 6153). The answers for both questions were combined in a way that "yes" was coded for every participant who ever chose "insulin", and "no" was coded if "insulin" was never chosen. Some participants had been asked multiple times for their age at first diagnosis of diabetes (ID 2976). Only the earliest available value for a participant was taken into account.

We generated a variable for self-reported type 1 diabetes (T1D) as follows from up to three timepoints: For participants who ever mentioned to have T1D (ID 20002, non-cancer illness code 1222 for type 1 diabetes), "yes" was coded. For participants, who never mentioned to have T1D, "no" was coded. Answers reported in the UKB repository as unclassifiable were coded as missing values.

ICD coded data on hospital admissions encompassing years 1981 to 1996 were retrieved from the Scottish Morbidity Record (SMR) as ICD version 9 (ICD9) codes (ID 41203 for main and ID 41205 for secondary diagnoses), data from 1996 onwards were retrieved from the Hospital Episode Statistics for England (HES) as ICD version 10 (ICD10) codes (ID 41202 for main and ID 41204 for secondary diagnoses). We generated a variable summarizing ICD10-coded T1D diagnoses from hospital admissions as follows: In case any main and/or secondary ICD10 code specific for T1D (codes: E10, E10.0 to E10.9) was reported in the UKB repository, "yes" was coded. In case only other ICD10 codes were reported, "no" was coded. Values were coded as missing, in case no ICD10 code was reported. ICD9-coded diagnoses from hospital admissions could be ignored, as no participant in our dataset obtained any main and/or secondary ICD9 code specific for juvenile diabetes (codes: 2501, 2501.1, 2502.1, 2509.1).

The primary T1D case definition (NDR-defined T1D) was built from the variables "age at diagnosis" and "insulin medication" as described in the main text. Four alternative T1D case definitions were designed, as also described in the main text. For the "weak" definition, controls were defined as all participants that had an age at diagnosis ≥ 30 years or answered "no" in at least three of the variables "insulin medication", "T1D (self-reported)", "T1D (ICD10)" and "age at diagnosis". For the "stringent" definition, controls were defined as those participants that had not reported to use insulin medication, those who reported insulin medication but had an age at diagnosis of ≥ 30 years, and those who reported insulin medication but did not start with insulin therapy within the first year of diagnosis.

1.3 Preparation of whole exome sequencing data

The HLA region is genetically very complex: Strong linkage disequilibrium (LD), frequent deviation from Hardy-Weinberg-equilibrium (HWE), high variation frequency (5.6 SNVs/kb in our dataset after removing singletons and doubletons) including many multi-allelic variants, copy-number variations and duplicate sequences (paralogs) complicate association analyses. In order to account for that, we did not filter for HWE, as many SNVs in the HLA region violate HWE (Graffelman et al., 2017;Kennedy et al., 2017), most likely due to evolutionary reasons, so that violation of HWE cannot directly be attributed to sequencing errors. None of the 49,025 participants in the dataset showed more than 10% missing SNV values, indicating that all participant's biological samples and gene sequencing results can be considered of high enough quality.

After filtering out monomorphic variants, 57807 SNVs remained for analysis. We did not exclude deletions, insertions and multi-allelic variants. Multi-allelic variants are coded in the way that every variant is separated into an individual SNV comparing to the reference allele resulting in missing values for the other alternative variations. We removed 37,276 singletons and doubletons, as genotyping errors (such as an error in the processing pipeline connected to duplicate sequence read marking reported by UKB) effecting these are especially likely to influence results. We removed 295 bi-allelic variants that showed >10% missing values. We performed standard quality control, but did not filter for HWE. Finally, we did not exclude SNVs with low minor allele frequencies (MAF), as we are interested in rare variants. The final dataset for analysis contained 20,236 SNVs for 49,025 unrelated participants. We used an additive coding of the genotypes: Homozygosity for the minor or major allele was coded as 0 or 2, respectively, heterozygosity was coded as 1.

1.4 Statistical analysis

All the statistical analyses were conducted in R (R Development Core Team, 2010), version 3.6.1. Quantile-quantile (QQ) and Manhattan plots were prepared using the R package qqman (Turner, 2014). Venn diagrams were prepared with the online tool InteractiVenn (Heberle et al., 2015).

Genetic PCs derived from UKB microarray data reflect on genetic variability and were included as covariates. Genetically determined sex was included as covariate as men have been reported to be at slightly higher risk to develop T1D in European populations (Maahs et al., 2010;Rawshani et al., 2018). Age was included as covariate as an association between genetic variation in the HLA region and age might be observed in case the analyzed SNVs impact survival, especially in a relatively old cohort such as UKB.

The single-SNV association tests were performed by evaluating $H_0\colon \beta_1 = 0$ in the following logistic regression model:

$$\log\left(odds_{T1D,NDR,i,j}\right) = \beta_0 + \beta_1 SNV_{ij} + \beta_2 age_i + \beta_3 sex_i + \sum_{k=1}^{10} \beta_{4,k} PC_{ik}$$

where j are SNVs 1, …, 20236, i are subjects 1, …, 49031 and k are PC 1, …, 10, assuming an additive model with 0, 1 or 2 copies of the minor allele on the log(odds) scale. For the analysis, the glm() function in R was used with default settings, performing a complete-case analysis.

Region-based SKAT was based on the logistic mixed model

$$\log\left(odds_{T1D,NDR,i}\right) = \alpha_0 + \alpha' X_i + \beta' G_i$$

where $X_i = (X_{i1}, \ldots, X_{i,12})$ is a matrix containing the 12 covariates as fixed effects (age, sex and top 10 genetic PCs) for subject i, and $G_i = (G_{i1}, \ldots, G_{ip})$ is a matrix containing the p SNVs as random effects in the gene of interest for subject i. The aggregated number of SNVs p depends on the region tested. We used the SKAT() function in the SKAT R package with default settings, which aggregates the effect of all SNVs in the tested region without cancelling effects with opposite direction, either through a linear kernel $K = GG'$ or through a linear-weighted kernel $K = GWW'G'$ with weights W.

In order to retain the multi-allelic SNVs with high missing rates, the missing cut-off was set to 1.0 in the SKAT function. Default imputation to the mean was used for missing values. The BiomaRt R package (Durinck et al., 2005;Durinck et al., 2009) was used to access Ensembl (release 99, January 2020) (Cunningham et al., 2019) to read out gene as well as exon boundaries in the extracted HLA region.

For the gene-based test, SNVs were aggregated for every one of the 148 protein-coding genes. The pseudogene LY6G6E had to be excluded from the analysis, as it did not contain any SNV, resulting in a set of 147 genes for the analysis. In order to condition on HLA-DQB1, the gene-based test was repeated with the linear kernel including the SNV 6:32660935:C:T as covariate in the null model. This SNV showed the second strongest association in the single variant analysis (Table 4). We did not condition on the most significant SNV coding for the HLA-DQB1 D57A variant as it is multi-allelic.

WES data as compared to whole genome sequencing or microarray data assesses variation at high coverage in protein coding regions. Thus, in combination with knowledge on protein function, they could facilitate uncovering physiological mechanisms based on functional protein variants involved in disease. However, in the UKB WES data, 45% of all the analyzed SNVs were not located in exons as based on exon boundaries. Thus, the gene-based approach might be dominated by SNVs in non-coding regions. In order to restrict the analysis to protein-coding variants, 1209 exons were analyzed in an exon-based SKAT test. For the exon-based test, SNVs were aggregated for every exon of every high-quality protein-coding transcript of protein-coding genes. As filter for high-quality transcripts, the GENCODE Basic flag was used, which is attached to all complete (in case no complete transcript is found, to the longest) protein-coding transcripts. In case there were exons with the same start or end position for a given gene, only the longest exon of these was included in the analysis. This resulted in 1646 exons, of which 1209 were available for analysis as they contained more than one SNV.

For quality control in the allele-based analysis, we set alleles with a maximum posterior probability of ≤0.7 (as described in (Bycroft et al., 2018)) and with combined maximum posterior probability for both alleles >1 and ≤1.4 to missing. This did not change any of the conclusions from the results as compared to the analysis of the raw dataset (data not shown).

2 Supplementary Figures and Tables

2.1 Supplementary Figures

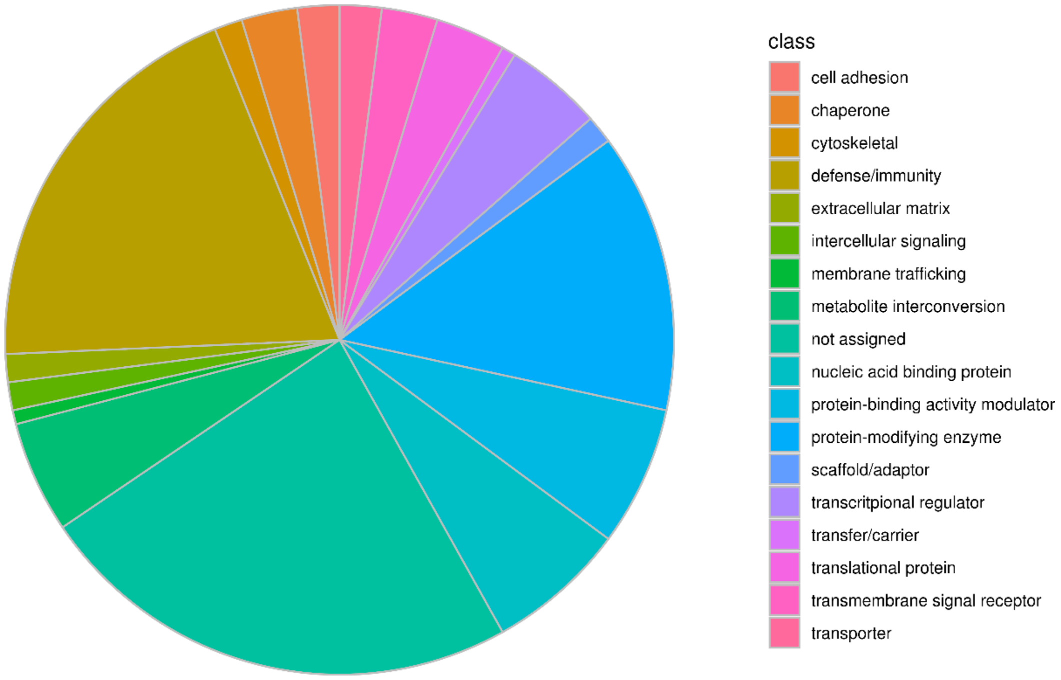

Supplementary Figure 1: The pie chart reflects the percentage of genes in the HLA region (chromosome 6, bases 29,722,775-33,314,387, genome build GRCh38/hg38) coding for proteins belonging to the given protein classes. PANTHER (Thomas et al., 2003) protein classes could be assigned to 112 of the 148 genes in the HLA region by PANTHER or based on UNIPROT entries.

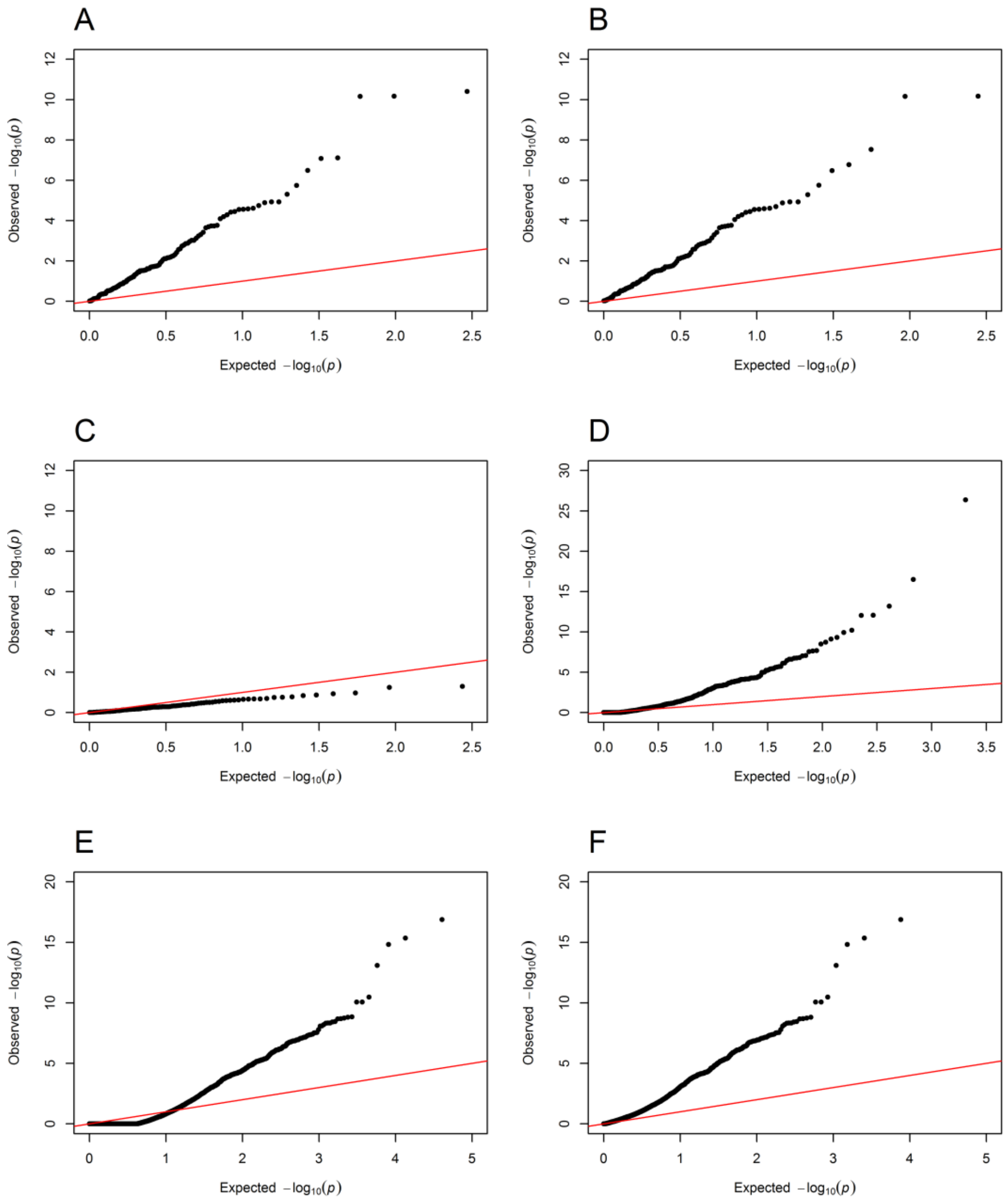

Supplementary Fig. 2: (**A**) Quantile-quantile (QQ) plot for the gene-based SKAT test (linear kernel) for association with NDR-defined T1D. (**B**) as (**A**) but limited to SNVs with a MAF > 0.01. By using a randomized outcome variable in (**C**), the results are in line with the null hypothesis. (**D**) QQ-plot for the exon-based SKAT test (linear model) for association with NDR-defined T1D. (**E**) QQ-plot for the single-SNV logistic regression analysis for association with NDR-defined T1D. (**F**) as in (**E**) but limited to the 3,825 SNVs with MAF > 0.01. This eliminates high p-values resulting from rare variants. At the same time low p-values become dominant with a median p-value of 0.27. The red line in the QQ-plots indicates values expected under the null hypothesis.

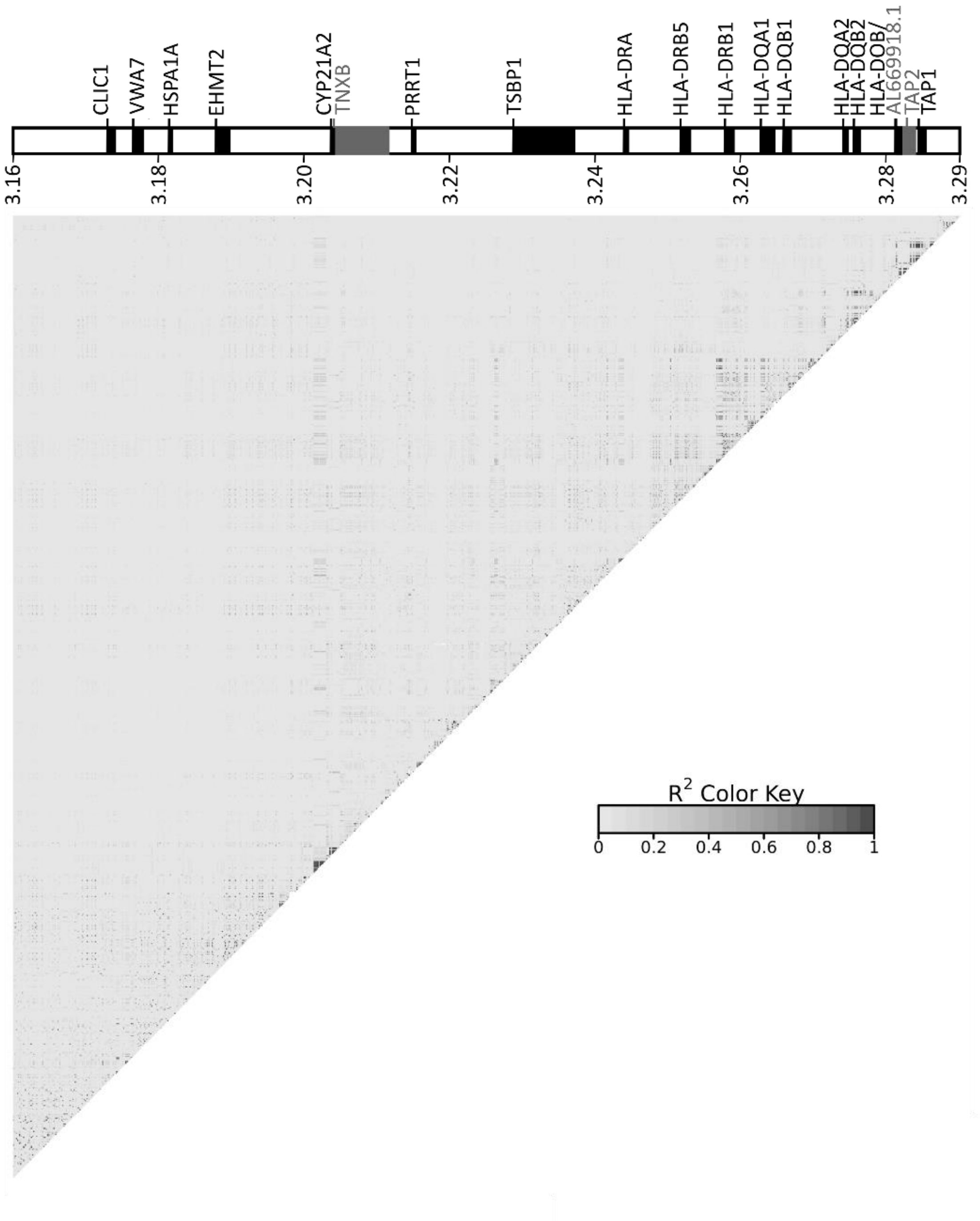

Supplementary Fig. 3: Plot of pairwise LD (measured as R^2) over all common (MAF >0.01) SNVs in exons in the region on chromosome 6 (31,600,000bp – 32,900,000bp) that shows the position of all genes associated in at least two tests above (in case of overlapping genes one of them is shown in grey). The gene axis label is in [$\cdot 10^7$bp]). The analysis was performed in R on the UK Biobank dataset.

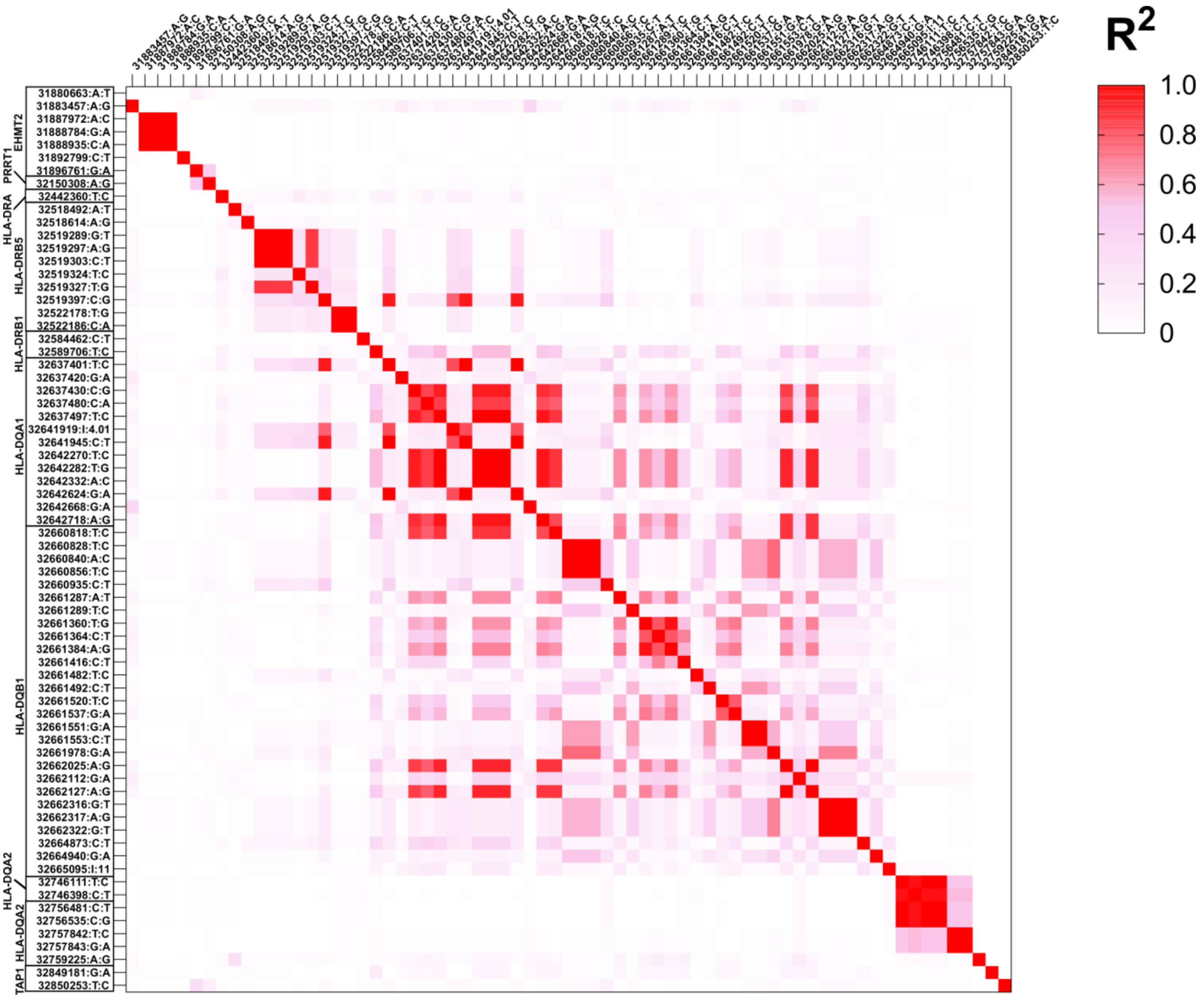

Supplementary Fig. 4: Plot of pairwise LD (measured as R^2) between SNVs in *EHMT2* that have p-values <0.05 and the SNVs found significantly associated with NDR-defined T1D in the single-SNV analysis. The analysis was performed with LDlink (Machiela and Chanock, 2015) for the British population of the 1000 Genomes Project using only analyzable bi-allelic SNVs.

2.2 Supplementary Tables

Supplementary Table 1: Demographic and clinical characteristics of NDR-defined T1D cases and controls of the analyzed sample of 49,025 participants from the UKB. Percentages in bracket are given per column.

	**control** (n=48,700)	**case** (n=97)	**overall** (n=49,025)
**age**			
mean (SEM)	56.6 (7.99)	53.5 (8.33)	56.6 (8.00)
median [min, max]	58.0 [39.0, 70.0]	53.0 [40.0, 69.0]	58.0 [39.0, 70.0]
**sex**			
female	26,532 (54.5%)	47 (48.5%)	26,684 (54.4%)
male	22,117 (45.4%)	50 (51.5%)	22,290 (45.5%)
missing	51 (0.1%)	0 (0%)	51 (0.1%)
**ethnicity**			
Caucasian	40,186 (82.5%)	82 (84.5%)	40,415 (82.4%)
missing	8,514 (17.5%)	15 (15.5%)	8,610 (17.6%)
**any diabetes**			
no	45,842 (94.1%)	0 (0%)	46,034 (93.9%)
yes	2,770 (5.7%)	97 (100%)	2,891 (5.9%)
missing	88 (0.2%)	0 (0%)	100 (0.2%)
**age at diagnosis**			
mean (SEM)	53.7 (10.2)	18.6 (6.51)	52.4 (12.0)
median [min, max]	55.0 [1.00, 76.0]	19.0 [1.00, 29.0]	55.0 [1.00, 76.0]
missing	46,097 (94.7%)	0 (0%)	46,317 (94.5%)
**insulin medication**			
no	48,287 (99.2%)	0 (0%)	48,287 (98.5%)
yes	413 (0.8%)	97 (100%)	534 (1.1%)
missing	0 (0%)	0 (0%)	204 (0.4%)
**insulin within first year**			
no	2,437 (5.0%)	17 (17.5%)	2,465 (5.0%)
yes	194 (0.4%)	80 (82.5%)	279 (0.6%)
missing	46069 (94.6%)	0 (0%)	46,281 (94.4%)
**T1D (self-reported)**			
no	40,202 (82.6%)	73 (75.3%)	40,450 (82.5%)
yes	23 (0.0%)	24 (24.7%)	47 (0.1%)
missing	8,475 (17.4%)	0 (0%)	8,528 (17.4%)
**T1D (ICD10)**			
no	42,010 (86.3%)	18 (18.6%)	42,232 (86.1%)
yes	270 (0.6%)	77 (79.4%)	355 (0.7%)
missing	6,420 (13.2%)	2 (2.1%)	6,438 (13.1%)

Supplementary Table 2: Demographic and clinical characteristics for the different T1D case definitions. Percentages in bracket are given per column.

	ICD10 (n=355)	weak (n=302)	NDR (n=97)	stringent (n=80)	self-report (n=47)
**age**					
mean (SEM)	58.0 (7.75)	57.4 (8.05)	53.5 (8.33)	52.7 (8.22)	53.6 (8.37)
median	59.0	59.0	53.0	52.5	55.0
[min. max]	[40.0. 70.0]	[40.0. 70.0]	[40.0. 69.0]	[40.0. 68.0]	[40.0. 69.0]
**sex**					
female	166 (46.8%)	146 (48.3%)	47 (48.5%)	41 (51.2%)	21 (44.7%)
male	189 (53.2%)	156 (51.7%)	50 (51.5%)	39 (48.8%)	26 (55.3%)
missing	0 (0%)	0 (0%)	0 (0%)	0 (0%)	0 (0%)
**ethnicity**					
Caucasian	281 (79.2%)	243 (80.5%)	82 (84.5%)	70 (87.5%)	42 (89.4%)
missing	74 (20.8%)	59 (19.5%)	15 (15.5%)	10 (12.5%)	5 (10.6%)
**any diabetes**					
no	30 (8.5%)	0 (0%)	0 (0%)	0 (0%)	0 (0%)
yes	325 (91.5%)	302 (100%)	97 (100%)	80 (100%)	47 (100%)
missing	0 (0%)	0 (0%)	0 (0%)	0 (0%)	0 (0%)
**age at diagnosis**					
mean (SEM)	38.6 (16.0)	35.4 (15.8)	18.6 (6.51)	17.6 (6.32)	30.5 (16.0)
median	43.0	40.0	19.0	17.0	29.0
[min. max]	[2.00. 72.0]	[1.00. 64.0]	[1.00. 29.0]	[1.00. 29.0]	[7.00. 59.0]
missing	37 (10.4%)	6 (2.0%)	0 (0%)	0 (0%)	0 (0%)
**insulin medication**					
no	79 (22.3%)	2 (0.7%)	0 (0%)	0 (0%)	3 (6.4%)
yes	274 (77.2%)	300 (99.3%)	97 (100%)	80 (100%)	44 (93.6%)
missing	2 (0.6%)	0 (0%)	0 (0%)	0 (0%)	0 (0%)
**insulin within first year**					
no	152 (42.8%)	112 (37.1%)	17 (17.5%)	0 (0%)	6 (12.8%)
yes	168 (47.3%)	185 (61.3%)	80 (82.5%)	80 (100%)	41 (87.2%)
missing	35 (9.9%)	5 (1.7%)	0 (0%)	0 (0%)	0 (0%)

Supplementary Table 3: List of genes found to be significantly associated with any of the T1D-definition by using gene-based SKAT, sorted by gene name. The start and end positions of the genes on chromosome 6 (genome build GRCh38/hg38) as derived from Ensembl (release 99, January 2020) (Cunningham et al., 2019) are given.

gene	geneID	start	end
ABHD16A	ENSG00000204427	31686955	31703356
AGER	ENSG00000204305	32180968	32184322
AL645922.1	ENSG00000244255	31927698	31952048
AL662899.3	ENSG00000204422	31686962	31714072
AL669918.1	ENSG00000250264	32813767	32838822
ATF6B	ENSG00000213676	32115264	32128253
ATP6V1G2	ENSG00000213760	31544462	31548427
ATP6V1G2-DDX39B	ENSG00000254870	31530219	31546608
BAG6	ENSG00000204463	31639028	31652705
BRD2	ENSG00000204256	32968594	32981505
BTNL2	ENSG00000204290	32393963	32407128
C6orf47	ENSG00000204439	31658298	31660778
CFB	ENSG00000243649	31945650	31952084
CLIC1	ENSG00000213719	31730581	31739763
CYP21A2	ENSG00000231852	32038327	32041644
DDAH2	ENSG00000213722	31727038	31730617
DDX39B	ENSG00000198563	31530219	31542448
DXO	ENSG00000204348	31969810	31972290
EHMT2	ENSG00000204371	31879759	31897687
GPANK1	ENSG00000204438	31661228	31666283
GPSM3	ENSG00000213654	32190766	32195523
HLA-DOB	ENSG00000241106	32812763	32820466
HLA-DQA1	ENSG00000196735	32628179	32647062
HLA-DQA2	ENSG00000237541	32741391	32747198
HLA-DQB1	ENSG00000179344	32659467	32668383
HLA-DQB2	ENSG00000232629	32756098	32763532
HLA-DRA	ENSG00000204287	32439878	32445046
HLA-DRB1	ENSG00000196126	32578769	32589848
HLA-DRB5	ENSG00000198502	32517353	32530287
HSPA1A	ENSG00000204389	31815543	31817946
LSM2	ENSG00000204392	31797396	31806966
MICB	ENSG00000204516	31494881	31511124
MSH5	ENSG00000204410	31739677	31762676
MSH5-SAPCD1	ENSG00000255152	31740020	31764851
NELFE	ENSG00000204356	31952087	31959038
NOTCH4	ENSG00000204301	32194843	32224067
PPT2	ENSG00000221988	32153441	32163678
PPT2-EGFL8	ENSG00000258388	32153845	32171978
PRRC2A	ENSG00000204469	31620715	31637771
PRRT1	ENSG00000204314	32148359	32153083

PSMB9	ENSG00000240065	32844136	32859851
RNF5	ENSG00000204308	32178405	32180793
TAP1	ENSG00000168394	32845209	32853978
TAP2	ENSG00000204267	32821833	32838770
TNXB	ENSG00000168477	32041153	32115334
TSBP1	ENSG00000204296	32288526	32371912
VARS1	ENSG00000204394	31777518	31795752
VWA7	ENSG00000204396	31765590	31777328
ZNRD1	ENSG00000066379	30058899	30064909

Supplementary Table 4: List of genes found to be significantly associated with NDR-defined T1D using the linear SKAT kernel sorted by p-value (same data as in Table 4) as compared to the results when restricting the analysis to SNVs with MAF > 0.01.

**gene**	**p-value (linear, NDR)**	**p-value (linear, NDR) MAF > 0.01**
PRRT1	$3.96 \cdot 10^{-11}$	<2 SNVs with MAF >0.01
HLA-DQA1	$6.77 \cdot 10^{-11}$	$6.75 \cdot 10^{-11}$
HLA-DQB1	$6.93 \cdot 10^{-11}$	$6.91 \cdot 10^{-11}$
HLA-DRB5	$7.72 \cdot 10^{-08}$	$2.94 \cdot 10^{-08}$
HLA-DQA2	$8.37 \cdot 10^{-08}$	$1.68 \cdot 10^{-07}$
HLA-DRB1	$3.24 \cdot 10^{-07}$	$3.31 \cdot 10^{-07}$
HLA-DQB2	$1.79 \cdot 10^{-06}$	$1.76 \cdot 10^{-06}$
HSPA1A	$4.93 \cdot 10^{-06}$	$5.16 \cdot 10^{-06}$
TSBP1	$1.17 \cdot 10^{-05}$	$1.19 \cdot 10^{-05}$
HLA-DRA	$1.18 \cdot 10^{-05}$	$1.18 \cdot 10^{-05}$
ABHD16A	$1.31 \cdot 10^{-05}$	$1.32 \cdot 10^{-05}$
CLIC1	$1.81 \cdot 10^{-05}$	$2.04 \cdot 10^{-05}$
AL669918.1	$2.46 \cdot 10^{-05}$	$2.46 \cdot 10^{-05}$
VWA7	$2.61 \cdot 10^{-05}$	$2.57 \cdot 10^{-05}$
MSH5	$2.76 \cdot 10^{-05}$	$2.77 \cdot 10^{-05}$
HLA-DOB	$2.82 \cdot 10^{-05}$	$2.79 \cdot 10^{-05}$
TAP2	$3.54 \cdot 10^{-05}$	$3.51 \cdot 10^{-05}$
MSH5-SAPCD1	$3.79 \cdot 10^{-05}$	$3.92 \cdot 10^{-05}$
PRRC2A	$5.22 \cdot 10^{-05}$	$5.19 \cdot 10^{-05}$
C6orf47	$6.35 \cdot 10^{-05}$	$6.25 \cdot 10^{-05}$
PSMB9	$7.98 \cdot 10^{-05}$	$8.97 \cdot 10^{-05}$
BRD2	$1.72 \cdot 10^{-04}$	$1.70 \cdot 10^{-04}$
TAP1	$1.82 \cdot 10^{-04}$	$1.81 \cdot 10^{-04}$
CYP21A2	$1.86 \cdot 10^{-04}$	$1.93 \cdot 10^{-04}$
LSM2	$2.01 \cdot 10^{-04}$	$2.00 \cdot 10^{-04}$
BTNL2	$2.28 \cdot 10^{-04}$	$2.29 \cdot 10^{-04}$

Supplementary Table 5: List of exons, found to be significantly associated with NDR-defined T1D, sorted by gene name. The exon ID and start and end position on chromosome 6 (genome build GRCh38/hg38) are given as derived from Ensembl (release 99, January 2020) (Cunningham et al., 2019).

exon ID	gene	exon_start	exon_end
ENSE00003589061	ABCF1	30590149	30590213
ENSE00003465858	AL669918.1	32829400	32829536
ENSE00003635259	BAG6	31641776	31641945
ENSE00001858405	CLIC1	31736262	31736567
ENSE00001656329	COL11A2	33167264	33167317
ENSE00003843744	CYP21A2	32040869	32041644
ENSE00003580154	EHMT2	31896606	31896824
ENSE00003786549	FLOT1	30730919	30731100
ENSE00001625708	HLA-DOB	32816861	32817002
ENSE00001619685	HLA-DPA1	33073471	33073669
ENSE00001911406	HLA-DPB1	33075990	33076141
ENSE00001766857	HLA-DQA1	32637401	32637540
ENSE00003658074	HLA-DQA1	32641972	32642253
ENSE00001465231	HLA-DQA1	32642610	32643017
ENSE00001614414	HLA-DQA2	32746240	32746414
ENSE00003421994	HLA-DQB1	32664798	32665067
ENSE00001596914	HLA-DQB1	32661967	32662248
ENSE00003562109	HLA-DQB1	32661347	32661457
ENSE00001703275	HLA-DQB2	32757773	32757883
ENSE00001723559	HLA-DQB2	32758850	32759131
ENSE00001663669	HLA-DQB2	32756098	32757304
ENSE00001775810	HLA-DQB2	32761660	32761926
ENSE00001930619	HLA-DRB1	32589643	32589848
ENSE00001731226	HLA-DRB1	32584109	32584378
ENSE00001801024	HLA-DRB1	32581557	32581838
ENSE00001641881	HLA-DRB5	32519370	32519651
ENSE00001768120	HLA-DRB5	32521905	32522174
ENSE00001715371	HLA-DRB5	32518556	32518666
ENSE00001690505	HSPA1A	31815543	31817942
ENSE00003703225	HSPA1A	31816327	31817946
ENSE00001691563	MICB	31506143	31506430
ENSE00001707124	PPP1R10	30606468	30606641
ENSE00001646872	PSMB8	32844279	32844679
ENSE00001727868	PSMB9	32858364	32858505
ENSE00003569101	SKIV2L	31961196	31961386
ENSE00001836503	SLC44A4	31863192	31863748
ENSE00003685114	TAP2	32829400	32829536
ENSE00001792039	TNXB	32047734	32048012
ENSE00003839368	TNXB	32041153	32041450
ENSE00003744412	TSBP1	32335912	32335934

ENSE00003725416	TSBP1	32293029	32293664
ENSE00003739283	TSBP1	32371694	32371912
ENSE00003463002	VARS2	30925274	30925385
ENSE00003555889	VWA7	31770001	31770113

Supplementary Table 6: List of 92 SNVs significantly associated after Bonferroni correction (p-value < 0.05/20,236 i.e. $2.47 \cdot 10^{-6}$) with NDR-defined T1D, sorted by p-value. The odds ratio with the 95% confidence interval (OR[95%CI]) is also given. The position in the genome is given in the form [chromosome number:position in bp:reference allele:minor allele]. Gene name and functional element of the variation are derived from dbSNP. In case of missense coding or synonymous variation in the functional protein domain the effected amino acid position assuming the remainder of the codon is reference sequence is given according to numbering of the PDB for mature proteins.

SNV	p-value	OR[95%CI]	MAF	gene	variant
6:32664911:T:G	$1.31 \cdot 10^{-17}$	3.26[2.49;4.28]	0.20	HLA-DQB1	D57A
6:32660935:C:T	$4.53 \cdot 10^{-16}$	3.07[2.34;4.02]	0.09	HLA-DQB1	intron
6:32661482:T:C	$1.47 \cdot 10^{-15}$	3.37[2.5;4.54]	0.10	HLA-DQB1	intron
6:32662112:G:A	$8.08 \cdot 10^{-14}$	3.25[2.38;4.42]	0.43	HLA-DQB1	synonymous A140A
6:32662322:G:T	$3.34 \cdot 10^{-11}$	2.08[1.68;2.59]	0.22	HLA-DQB1	intron
6:32662317:A:G	$8.55 \cdot 10^{-11}$	2.05[1.65;2.55]	0.23	HLA-DQB1	intron
6:32662316:G:T	$8.66 \cdot 10^{-11}$	2.05[1.65;2.55]	0.23	HLA-DQB1	intron
6:30911339:C:G	$1.46 \cdot 10^{-09}$	162.01 [31.16;842.29]	0.00008	GTF2H4	intron
6:32637430:C:G	$1.52 \cdot 10^{-09}$	2.39[1.8;3.18]	0.44	HLA-DQA1	5'UTR
6:32637480:C:A	$1.84 \cdot 10^{-09}$	2.37[1.79;3.13]	0.46	HLA-DQA1	signal peptide
6:32530209:G:A	$2.03 \cdot 10^{-09}$	4.99[2.95;8.45]	0.02	HLA-DRB5	signal peptide
6:32660840:A:C	$2.16 \cdot 10^{-09}$	2.37[1.79;3.15]	0.15	HLA-DQB1	intron
6:32589618:C:G	$3.54 \cdot 10^{-09}$	2.33[1.76;3.09]	0.08	HLA-DRB1	intron
6:32660856:T:C	$3.79 \cdot 10^{-09}$	2.35[1.77;3.13]	0.16	HLA-DQB1	intron
6:32522186:C:A	$4.60 \cdot 10^{-09}$	2.03[1.6;2.57]	0.16	HLA-DRB5	intron
6:32641945:C:T	$4.72 \cdot 10^{-09}$	2.34[1.76;3.11]	0.18	HLA-DQA1	intron
6:32519289:G:T	$4.86 \cdot 10^{-09}$	1.98[1.57;2.48]	0.19	HLA-DRB5	intron
6:32661276:C:G	$6.33 \cdot 10^{-09}$	2.33[1.75;3.11]	0.45	HLA-DQB1	intron
6:32150308:A:G	$7.94 \cdot 10^{-09}$	6.1[3.3;11.28]	0.01	PRRT1	intron
6:32757908:T:C	$8.50 \cdot 10^{-09}$	3.77[2.4;5.91]	0.04	HLA-DQB2	intron
6:32660828:T:C	$1.62 \cdot 10^{-08}$	2.3[1.72;3.07]	0.14	HLA-DQB1	intron
6:32522178:T:G	$2.79 \cdot 10^{-08}$	2.16[1.64;2.83]	0.19	HLA-DRB5	intron
6:32642332:A:C	$2.87 \cdot 10^{-08}$	2.23[1.68;2.97]	0.48	HLA-DQA1	intron
6:32637497:T:C	$3.00 \cdot 10^{-08}$	2.24[1.68;2.97]	0.49	HLA-DQA1	synonymous/ signal peptide
6:32641919:I:4.01	$3.80 \cdot 10^{-08}$	2.15[1.64;2.82]	0.07	HLA-DQA1	intron
6:32661416:C:T	$4.18 \cdot 10^{-08}$	2.6[1.85;3.66]	0.59	HLA-DQB1	transmembrane
6:32519327:T:G	$4.38 \cdot 10^{-08}$	1.82[1.47;2.25]	0.27	HLA-DRB5	intron
6:32519297:A:G	$4.60 \cdot 10^{-08}$	1.84[1.48;2.29]	0.22	HLA-DRB5	intron
6:32642270:T:C	$5.12 \cdot 10^{-08}$	2.44[1.77;3.37]	0.56	HLA-DQA1	intron

6:32664940:G:A	$5.82 \cdot 10^{-08}$	1.93[1.52;2.45]	0.25	HLA-DQB1	synonymous/ Y47Y
6:32665095:I:11	$7.00 \cdot 10^{-08}$	2.28[1.69;3.08]	0.31	HLA-DQB1	intron
6:32664971:T:A	$7.04 \cdot 10^{-08}$	2.08[1.59;2.71]	0.13	HLA-DQB1	Y37F
6:32522152:C:T	$7.49 \cdot 10^{-08}$	1.9[1.5;2.4]	0.17	HLA-DRB5	synonymous
6:32522151:A:G	$8.19 \cdot 10^{-08}$	1.9[1.5;2.39]	0.17	HLA-DRB5	Y13D
6:32642282:T:G	$8.33 \cdot 10^{-08}$	2.42[1.75;3.34]	0.56	HLA-DQA1	intron
6:32664972:A:T	$8.79 \cdot 10^{-08}$	2.07[1.58;2.7]	0.13	HLA-DQB1	Y37N
6:32637401:T:C	$9.80 \cdot 10^{-08}$	2.19[1.64;2.93]	0.11	HLA-DQA1	upstream
6:32661364:C:T	$1.11 \cdot 10^{-07}$	2.57[1.81;3.64]	0.60	HLA-DQB1	cytoplasmic
6:32746111:T:C	$1.14 \cdot 10^{-07}$	0.42[0.31;0.58]	0.46	HLA-DQA2	intron
6:32661360:T:G	$1.17 \cdot 10^{-07}$	2.57[1.81;3.64]	0.60	HLA-DQB1	cytoplasmic
6:32661289:T:C	$1.26 \cdot 10^{-07}$	2.22[1.65;2.99]	0.15	HLA-DQB1	intron
6:32662158:C:G	$1.33 \cdot 10^{-07}$	2.41[1.74;3.35]	0.57	HLA-DQB1	A125G
6:32580955:C:A	$1.42 \cdot 10^{-07}$	2.94[1.97;4.38]	0.09	HLA-DRB1	intron
6:32756535:C:G	$1.42 \cdot 10^{-07}$	0.42[0.31;0.58]	0.46	HLA-DQB2	intron
6:32519324:T:C	$1.44 \cdot 10^{-07}$	1.79[1.44;2.23]	0.26	HLA-DRB5	intron
6:32662025:A:G	$1.50 \cdot 10^{-07}$	2.56[1.8;3.64]	0.61	HLA-DQB1	synonymous/ D169D
6:32661384:A:G	$1.63 \cdot 10^{-07}$	2.56[1.8;3.64]	0.61	HLA-DQB1	synonymous transmembrane
6:32519303:C:T	$1.69 \cdot 10^{-07}$	1.79[1.44;2.23]	0.23	HLA-DRB5	intron
6:32662127:A:G	$1.78 \cdot 10^{-07}$	2.5[1.77;3.52]	0.60	HLA-DQB1	synonymous/ D135D
6:32642668:G:A	$1.86 \cdot 10^{-07}$	2.2[1.64;2.97]	0.17	HLA-DQA1	synonymous/ transmembrane
6:32746398:C:T	$1.98 \cdot 10^{-07}$	0.42[0.3;0.58]	0.45	HLA-DQA2	3'UTR
6:32661287:A:T	$2.19 \cdot 10^{-07}$	2.28[1.67;3.12]	0.54	HLA-DQB1	intron
6:33202144:G:A	$2.21 \cdot 10^{-07}$	58.7 [12.58;273.98]	0.0002	SLC39A7/ RXRB	intron/ upstream
6:32589588:A:C	$2.50 \cdot 10^{-07}$	2.1[1.59;2.79]	0.09	HLA-DRB1	intron
6:32642624:G:A	$3.33 \cdot 10^{-07}$	2.14[1.6;2.87]	0.19	HLA-DQA1	A184T
6:32757842:T:C	$3.78 \cdot 10^{-07}$	0.37[0.25;0.54]	0.39	HLA-DQB2	tail
6:32661978:G:A	$3.87 \cdot 10^{-07}$	2.18[1.61;2.94]	0.17	HLA-DQB1	T185I
6:32850253:T:C	$3.98 \cdot 10^{-07}$	3.6[2.19;5.91]	0.03	TAP1	intron
6:32584170:D:1	$4.03 \cdot 10^{-07}$	2.34[1.68;3.25]	0.25	HLA-DRB1	frame shift A74
6:32757843:G:A	$4.42 \cdot 10^{-07}$	0.37[0.25;0.54]	0.38	HLA-DQB2	synonymous
6:32757848:T:C	$4.49 \cdot 10^{-07}$	0.37[0.25;0.54]	0.38	HLA-DQB2	tail
6:32664992:T:C	$5.44 \cdot 10^{-07}$	1.98[1.52;2.59]	0.16	HLA-DQB1	Y30C
6:32661553:C:T	$5.55 \cdot 10^{-07}$	2.13[1.58;2.86]	0.17	HLA-DQB1	intron
6:32756481:C:T	$6.22 \cdot 10^{-07}$	0.45[0.32;0.61]	0.45	HLA-DQB2	intron
6:32522163:G:C	$6.38 \cdot 10^{-07}$	2.21[1.62;3.02]	0.53	HLA-DRB5	Q9E
6:32166601:C:T	$6.50 \cdot 10^{-07}$	42.44 [9.69;185.75]	0.0002	EGFL8	R69C
6:32661551:G:A	$6.59 \cdot 10^{-07}$	2.12[1.58;2.85]	0.17	HLA-DQB1	intron
6:32661537:G:A	$6.63 \cdot 10^{-07}$	2.33[1.67;3.26]	0.59	HLA-DQB1	intron

6:32522205:G:A	$7.59 \cdot 10^{-07}$	1.95[1.5;2.54]	0.10	HLA-DRB5	intron
6:32660818:T:C	$7.69 \cdot 10^{-07}$	2.14[1.58;2.9]	0.54	HLA-DQB1	intron
6:32442360:T:C	$7.82 \cdot 10^{-07}$	3.57[2.16;5.92]	0.75	HLA-DRA	intron
6:32758995:G:A	$7.87 \cdot 10^{-07}$	2.83[1.87;4.28]	0.09	HLA-DQB2	synonymous
6:32746405:D:1	$7.95 \cdot 10^{-07}$	0.43[0.3;0.6]	0.46	HLA-DQA2	3'UTR
6:32661492:C:T	$7.99 \cdot 10^{-07}$	2.09[1.56;2.8]	0.18	HLA-DQB1	intron
6:32849181:G:A	$8.37 \cdot 10^{-07}$	2.48[1.73;3.57]	0.65	TAP1	intron
6:32664998:G:C	$9.53 \cdot 10^{-07}$	1.96[1.5;2.56]	0.17	HLA-DQB1	T28S
6:32661520:T:C	$9.94 \cdot 10^{-07}$	2.39[1.68;3.38]	0.62	HLA-DQB1	intron
6:32759225:A:G	$1.01 \cdot 10^{-06}$	2.84[1.87;4.31]	0.20	HLA-DQB2	intron
6:32664976:C:T	$1.11 \cdot 10^{-06}$	1.95[1.49;2.55]	0.13	HLA-DQB1	synonymous E35E
6:32042508:C:T	$1.16 \cdot 10^{-06}$	348.09 [32.91;3681.99]	0.00004	TNXB	E4051K
6:32637420:G:A	$1.17 \cdot 10^{-06}$	2.14[1.57;2.91]	0.09	HLA-DQA1	5'UTR
6:32642718:A:G	$1.23 \cdot 10^{-06}$	2[1.51;2.64]	0.51	HLA-DQA1	tail
6:32519738:C:T	$1.30 \cdot 10^{-06}$	2.43[1.7;3.49]	0.04	HLA-DRB5	intron
6:32518492:A:T	$1.32 \cdot 10^{-06}$	2.26[1.62;3.14]	0.09	HLA-DRB5	intron
6:32584462:C:T	$1.43 \cdot 10^{-06}$	1.96[1.49;2.58]	0.08	HLA-DRB1	intron
6:32518614:A:G	$1.49 \cdot 10^{-06}$	2.16[1.58;2.96]	0.25	HLA-DRB5	synonymous
6:32522190:G:C	$1.66 \cdot 10^{-06}$	1.79[1.41;2.27]	0.30	HLA-DRB5	intron
6:32519397:C:G	$1.70 \cdot 10^{-06}$	2.27[1.62;3.17]	0.15	HLA-DRB5	V180L
6:32664873:C:T	$2.01 \cdot 10^{-06}$	1.85[1.43;2.38]	0.45	HLA-DQB1	G70R
6:32660791:A:G	$2.04 \cdot 10^{-06}$	2.08[1.54;2.82]	0.09	HLA-DQB1	intron
6:32530100:C:G	$2.13 \cdot 10^{-06}$	2.58[1.74;3.81]	0.08	HLA-DRB5	intron
6:32589706:T:C	$2.31 \cdot 10^{-06}$	2.74[1.81;4.17]	0.70	HLA-DRB1	signal peptide

Supplementary Table 7: List of HLA alleles found to be significantly associated (p-value < 0.05/362) with NDR-defined T1D, sorted by p-value. The odds ratio (OR) with the 95% confidence interval (95%CI) is also given.

HLA allele	p-value	OR [95%CI]
DQB1*0302	$3.42 \cdot 10^{-16}$	3.6 [2.65;4.91]
DRB1*0401	$1.13 \cdot 10^{-08}$	2.66 [1.9;3.73]
DQB1*0201	$2.55 \cdot 10^{-08}$	2.41 [1.77;3.28]
DQA1*0301	$3.69 \cdot 10^{-08}$	2.29 [1.7;3.07]
DRB1*0301	$5.25 \cdot 10^{-08}$	2.37 [1.74; 3.23]
DRB5*9901	$2.80 \cdot 10^{-05}$	5.8 [2.55; 13.19]
A*0302	$5.46 \cdot 10^{-05}$	12.84 [3.72; 44.34]
DRB5*0101	$7.28 \cdot 10^{-05}$	0.19 [0.09; 0.43]
DQB1*0602	$1.33 \cdot 10^{-04}$	0.22 [0.1; 0.48]

2.3 Supplementary References

Bycroft, C., Freeman, C., Petkova, D., Band, G., Elliott, L.T., Sharp, K., Motyer, A., Vukcevic, D., Delaneau, O., O'connell, J., Cortes, A., Welsh, S., Young, A., Effingham, M., Mcvean, G., Leslie, S., Allen, N., Donnelly, P., and Marchini, J. (2018). The UK Biobank resource with deep phenotyping and genomic data. *Nature* 562**,** 203-209.

Cunningham, F., Achuthan, P., Akanni, W., Allen, J., Amode, M.R., Armean, I.M., Bennett, R., Bhai, J., Billis, K., Boddu, S., Cummins, C., Davidson, C., Dodiya, K.J., Gall, A., Giron, C.G., Gil, L., Grego, T., Haggerty, L., Haskell, E., Hourlier, T., Izuogu, O.G., Janacek, S.H., Juettemann, T., Kay, M., Laird, M.R., Lavidas, I., Liu, Z., Loveland, J.E., Marugan, J.C., Maurel, T., Mcmahon, A.C., Moore, B., Morales, J., Mudge, J.M., Nuhn, M., Ogeh, D., Parker, A., Parton, A., Patricio, M., Abdul Salam, A.I., Schmitt, B.M., Schuilenburg, H., Sheppard, D., Sparrow, H., Stapleton, E., Szuba, M., Taylor, K., Threadgold, G., Thormann, A., Vullo, A., Walts, B., Winterbottom, A., Zadissa, A., Chakiachvili, M., Frankish, A., Hunt, S.E., Kostadima, M., Langridge, N., Martin, F.J., Muffato, M., Perry, E., Ruffier, M., Staines, D.M., Trevanion, S.J., Aken, B.L., Yates, A.D., Zerbino, D.R., and Flicek, P. (2019). Ensembl 2019. *Nucleic Acids Res* 47**,** D745-D751.

Durinck, S., Moreau, Y., Kasprzyk, A., Davis, S., De Moor, B., Brazma, A., and Huber, W. (2005). BioMart and Bioconductor: a powerful link between biological databases and microarray data analysis. *Bioinformatics* 21**,** 3439-3440.

Durinck, S., Spellman, P.T., Birney, E., and Huber, W. (2009). Mapping identifiers for the integration of genomic datasets with the R/Bioconductor package biomaRt. *Nat Protoc* 4**,** 1184-1191.

Fry, A., Littlejohns, T.J., Sudlow, C., Doherty, N., Adamska, L., Sprosen, T., Collins, R., and Allen, N.E. (2017). Comparison of Sociodemographic and Health-Related Characteristics of UK Biobank Participants With Those of the General Population. *Am J Epidemiol* 186**,** 1026-1034.

Graffelman, J., Jain, D., and Weir, B. (2017). A genome-wide study of Hardy-Weinberg equilibrium with next generation sequence data. *Hum Genet* 136**,** 727-741.

Heberle, H., Meirelles, G.V., Da Silva, F.R., Telles, G.P., and Minghim, R. (2015). InteractiVenn: a web-based tool for the analysis of sets through Venn diagrams. *BMC Bioinformatics* 16**,** 169.

Kennedy, A.E., Ozbek, U., and Dorak, M.T. (2017). What has GWAS done for HLA and disease associations? *Int J Immunogenet* 44**,** 195-211.

Keyes, K.M., and Westreich, D. (2019). UK Biobank, big data, and the consequences of non-representativeness. *Lancet* 393**,** 1297.

Maahs, D.M., West, N.A., Lawrence, J.M., and Mayer-Davis, E.J. (2010). Epidemiology of type 1 diabetes. *Endocrinol Metab Clin North Am* 39**,** 481-497.

Machiela, M.J., and Chanock, S.J. (2015). LDlink: a web-based application for exploring population-specific haplotype structure and linking correlated alleles of possible functional variants. *Bioinformatics* 31**,** 3555-3557.

R Development Core Team (2010). "R: A language and environment for statistical computing". (Vienna, Austria: R Foundation for Statistical Computing).

Rawshani, A., Sattar, N., Franzen, S., Rawshani, A., Hattersley, A.T., Svensson, A.M., Eliasson, B., and Gudbjornsdottir, S. (2018). Excess mortality and cardiovascular disease in young adults

with type 1 diabetes in relation to age at onset: a nationwide, register-based cohort study. *Lancet* 392**,** 477-486.

Thomas, P.D., Campbell, M.J., Kejariwal, A., Mi, H., Karlak, B., Daverman, R., Diemer, K., Muruganujan, A., and Narechania, A. (2003). PANTHER: a library of protein families and subfamilies indexed by function. *Genome Res* 13**,** 2129-2141.

Turner, S.D. (2014). qqman: an R package for visualizing GWAS results using Q-Q and manhattan plots. *bioRxiv*.

Van Hout, C.V., Tachmazidou, I., Backman, J.D., Hoffman, J.X., Ye, B., Pandey, A.K., Gonzaga-Jauregui, C., Khalid, S., Liu, D., Banerjee, N., Li, A.H., Colm, O.D., Marcketta, A., Staples, J., Schurmann, C., Hawes, A., Maxwell, E., Barnard, L., Lopez, A., Penn, J., Habegger, L., Blumenfeld, A.L., Yadav, A., Praveen, K., Jones, M., Salerno, W.J., Chung, W.K., Surakka, I., Willer, C.J., Hveem, K., Leader, J.B., Carey, D.J., Ledbetter, D.H., Cardon, L., Yancopoulos, G.D., Economides, A., Coppola, G., Shuldiner, A.R., Balasubramanian, S., Cantor, M., Nelson, M.R., Whittaker, J., Reid, J.G., Marchini, J., Overton, J.D., Scott, R.A., Abecasis, G., Yerges-Armstrong, L., and Baras, A. (2019). Whole exome sequencing and characterization of coding variation in 49,960 individuals in the UK Biobank. *bioRxiv***,** 572347.
